# Natural hydrogen in the volcanic-bearing sedimentary basin: Origin, conversion, and production rates

**DOI:** 10.1126/sciadv.adr6771

**Published:** 2025-01-24

**Authors:** Quanyou Liu, Yongbo Wei, Pengpeng Li, Xiaowei Huang, Qingqiang Meng, Xiaoqi Wu, Dongya Zhu, Huiyuan Xu, Yin Fu, Di Zhu, Wang Zhang, Zhijun Jin

**Affiliations:** ^1^ Institute of Energy, School of Earth and Space Sciences, Peking University, Beijing 100871, China.; ^2^ Northwest Institute of Eco-Environment and Resources, Chinese Academy of Sciences, Lanzhou 73000, China.; ^3^ Key Laboratory of Deep Petroleum Intelligent Exploration and Development, Institute of Geology and Geophysics, Chinese Academy of Sciences, Beijing 100029, China.; ^4^ University of Chinese Academy of Sciences, Beijing 100049, China.; ^5^ Petroleum Exploration and Production Research Institute, SINOPEC, Beijing 100083, China.; ^6^ School of Sustainable Energy and Resources, Nanjing University, Suzhou 215163, China.; ^7^ Suzhou Grand Energy Technology Co. Ltd., Suzhou 215010, China.

## Abstract

The origins of natural hydrogen in natural gas systems of sedimentary basins and the capacity of these systems to store hydrogen remain inadequately understood, posing crucial questions for the large-scale exploration of natural hydrogen. This study reports on the natural gas composition, stable carbon and hydrogen isotopic values, and helium isotopic values of gas samples collected from the Qingshen gas deposit within volcanic rocks of the Songliao Basin. Natural hydrogen primarily originates from water radiolysis, water-rock interactions (WRI), and mantle. The Qingshen gas deposit contains 95.23 × 10^9^ cubic meters of abiotic CH_4_, of which 15.24 × 10^9^ cubic meters was generated through hydrogen conversion via Fischer-Tropsch synthesis, with the maximum original hydrogen reserves calculated to be approximately 61.9 × 10^9^ cubic meters. We estimated that the study area has generated a maximum total of 572 × 10^9^ cubic meters of radiolytic hydrogen, 248 × 10^9^ cubic meters of WRI hydrogen, and 127 × 10^9^ cubic meters of mantle-derived hydrogen.

## Teaser

Sedimentary basins could store natural hydrogen within natural gas systems.

## INTRODUCTION

In recent years, escalating global energy demands coupled with mounting environmental concerns have propelled hydrogen into the spotlight as an efficient and clean energy source (*1*). Although some believe that the prospects for hydrogen use across various industries may be poor (*2*), there is nevertheless a current need for clean hydrogen to replace gray or polluting hydrogen (steam reforming of methane). Natural hydrogen extracted directly from geological formations, a clean and economical method of hydrogen production, has been dubbed “golden hydrogen” or “white hydrogen” (*3*, *4*). “Hunt for natural hydrogen heats up” was recognized by *Science* as one of the “2023 Breakthroughs of the Year,” underscoring its significance as a monumental scientific discovery and a pivotal trend for the future.

The geological formations of the natural world contain vast reserves of natural hydrogen, with the Precambrian continental lithosphere alone estimated to generate approximately 554 million tons of hydrogen annually (*5*). To date, the global scientific community has identified hundreds of natural hydrogen occurrences (*6*–*11*). In the context of natural hydrogen discoveries, the dominance of inorganic sources is evident, and high concentrations of natural hydrogen were primarily found in ore deposits, ophiolite belts, and deep basement rocks (*12*, *13*). For example, in Albania’s Bulqizë ophiolite, degassing of serpentinized chromitite has been observed to release gas comprising up to 84% H_2_, with an annual release estimated at no less than 200 tons (100 × 10^6^ mol/year) (*9*).

Theoretically, the internal strata of sedimentary basins feature various traps capable of storing natural gas, suggesting a high likelihood of natural hydrogen being similarly trapped. This process is similar to that of petroleum and natural gas systems and can lead to the formation of hydrogen-rich natural gas reservoirs (*14*–*16*). However, the propensity for considerable natural hydrogen dissipation is attributed to the small size, high reactivity, and ease of diffusion of hydrogen molecules (*3*). Compared to regions with high concentrations of natural hydrogen, natural hydrogen concentrations in clastic rocks, volcanic rocks, and carbonate reservoirs within sedimentary basins are generally lower. The low concentration and reserves of geological hydrogen in geological structures present notable limitations for commercial exploitation, and it remains to be proven that proposed development projects can produce sufficient marketable quantities to attract potential buyers. Knowledge of natural hydrogen and efforts to develop the resource are still in the exploratory stages worldwide, with only the natural hydrogen reservoir in Mali, West Africa having been commercially developed (*17*). Natural hydrogen in sedimentary basins may have multiple origins (organic and inorganic or crustal and mantle) and undergo complex conversion processes (*11*, *12*, *18*–*22*). Therefore, in the complex geological settings of sedimentary basins, establishing and quantitatively evaluating the various origins and conversion processes of natural hydrogen in natural gas reservoirs are vital for discovering hydrogen-rich natural gas reservoirs. In addition, assessing the production rates of natural hydrogen from different origins and the store rates of natural hydrogen by natural gas reservoir systems will be instrumental in future natural hydrogen exploration in global sedimentary basins.

**Fig. 1.**
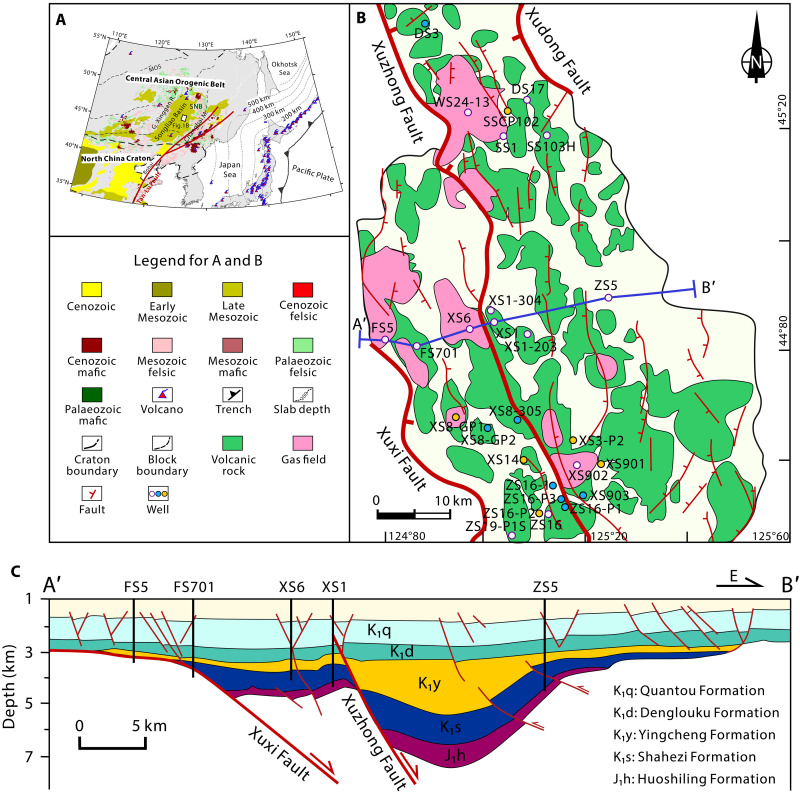
Location and geological context for the study area. (**A**) Tectonics of East Asia and Songliao Basin, modified after (*23*). SNB, Songnen Block; MOS, Mongol-Okhotsk Suture. The yellow circles represent group I gases, while the blue circles represent group II gases. (**B**) Distribution of volcanic rocks and gas deposits of the Yingcheng Formation from the Xujiaweizi fault depression in the northern Songliao Basin, modified after (*75*). (**C**) Structural cross section of the Xujiaweizi rift depression, modified after (*76*).

The Songliao Basin is a continental Mesozoic-Cenozoic sedimentary basin located in Northeast China. The Songliao Basin dominates the eastern part of the Songnen Block (Fig. 1A), which is a lithospheric block located within the eastern segment of the Central Asian Orogenic Belt. A collisional assemblage of microcontinents and multiple periods of magmatic activity occurred across this region during the development of the Palaeo-Asian Ocean subduction system in the late Palaeozoic [∼350 to 250 million years (Ma)], the Mongolia-Okhotsk Ocean subduction system in the early Mesozoic (∼250 to 200 Ma), and the (Palaeo-)Pacific Plate subduction system in the Meso-Cenozoic (<200 Ma) (*23*). The Qingshen gas deposit, the world’s largest deep volcanic rock gas deposit, is located in the Xujiaweizi rift depression of the Songliao Basin (Fig. 1B) (*24*). The rift system is characterized by thick volcanic and clastic strata, including the Upper Jurassic Huoshiling Formation and the Lower Cretaceous Shahezi, Yingcheng, Denglouku, and Quantou Formations (Fig. 1C), with deep natural gas primarily accumulating in the volcanic rocks. The Songke 2 (SK2) well, located in the Xujiahuizi fault depression, is the world’s first continental scientific drilling well to penetrate Cretaceous continental strata. It has revealed extensive continuous hydrogen gas anomalies in the Lower Cretaceous Denglouku and Yingcheng Formations and the basement rocks, with hydrogen concentrations ranging from 1.36 to 26.89% (*25*). Although the discovery of high concentrations of natural hydrogen during the drilling of the SK2 well demonstrated the hydrogen exploration potential of the deep formations in the Songliao Basin, the origin and conversion processes of hydrogen in industrial natural gas reservoirs require further investigation.

We obtained natural gas samples from 23 wells in the Qingshen gas deposit within the Xujiaweizi depression. On the basis of gas composition and carbon-hydrogen-helium isotope values (table S1), a crust-mantle mixed origin and conversion system for hydrogen in the volcanic-bearing sedimentary basin was established. We provided estimates of hydrogen production rates in the volcanic-bearing sedimentary basin and quantitatively assessed the natural gas system’s ability to store natural hydrogen. This holds substantial implications for discovering and exploring natural hydrogen in sedimentary basins.

## RESULTS

### Characterization of natural gas composition

**Fig. 2.**
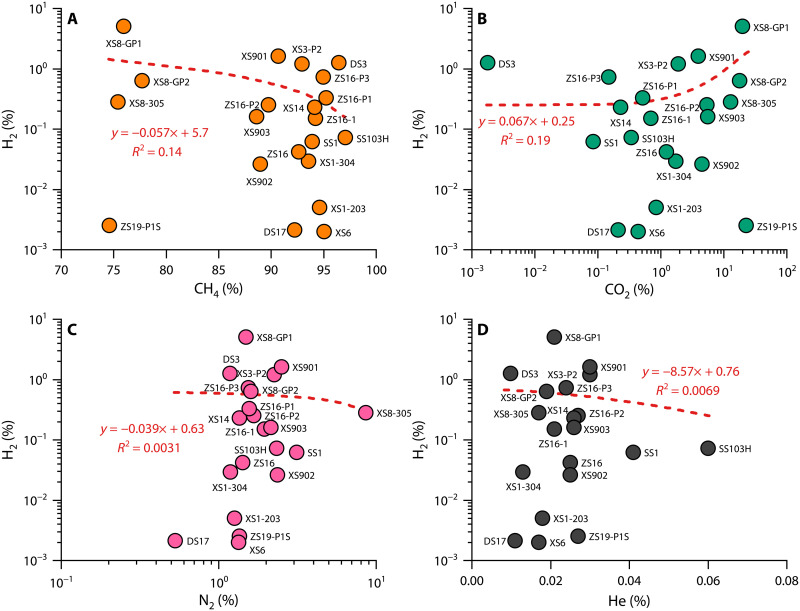
Correlation of hydrogen concentration with CH_4_, CO_2_, N_2_, and He concentrations for the Qingshen gas deposit, Songliao Basin. (**A**) Hydrogen concentration versus CH_4_ concentration. (**B**) Hydrogen concentration versus CO_2_ concentration. (**C**) Hydrogen concentration versus N_2_ concentration. (**D**) Hydrogen concentration versus He concentration.

The 23 gas samples we examined exhibit variable molecular composition (table S1), with CH_4_ content ranging from 74.61 to 97.09% and CO_2_ content ranging from 0.0018 to 22.68%. N_2_ and He contents range from 0.53 to 8.61% and 99 to 600 parts per million (ppm), respectively. The concentration of natural hydrogen is relatively low compared to Mali and Kansas (*7*, *17*), ranging from 0 to 5.0%, with an average of 0.53%. The gas compositions of the Qingshen gas deposit within the Songliao Basin indicate that natural hydrogen primarily occurs in natural gas reservoirs dominated by CH_4_, CO_2_, and N_2_. The weak correlation between hydrogen concentration and the contents of CH_4_, N_2_, CO_2_, and He suggests that natural hydrogen does not have an identical genetic link to the four gases and may undergo a complex process of generation and conversion (Fig. 2).

### Characterization of gas isotopes

We conducted carbon, hydrogen, and helium isotope testing experiments on the gas samples to better understand the origins of various gas components. The results show that δ^13^C_1_, δ^13^C_2_, and δ^13^C_3_ values range between −32.5 and −25.0 per mil (‰) (averaging −27.8‰), −34.8 and −21.3‰ (averaging −30.7‰), and −34.8 and −20.8‰ (averaging −31.1‰), respectively. The differences in δ^13^C among the C_1_-C_3_ alkanes (methane through propane) in some of the samples follow an “inverse” trend (fig. S1), i.e., δ^13^C_1_ > δ^13^C_2_ > δ^13^C_3_, or opposite the usual pattern of increasing δ^13^C with increasing carbon number (*26*). This carbon isotopic inverse feature may arise from factors including but not limited to the high maturity of the source rocks, cracking of heavy hydrocarbons, and mixing of gases from different sources (*27*). The δ^13^C-CO_2_ values range from −13.2 to −3.9‰, with several samples exhibiting heavier isotopic characteristics (>−10.0‰), suggesting an inorganic origin for CO_2_ (*28*).

**Fig. 3.**
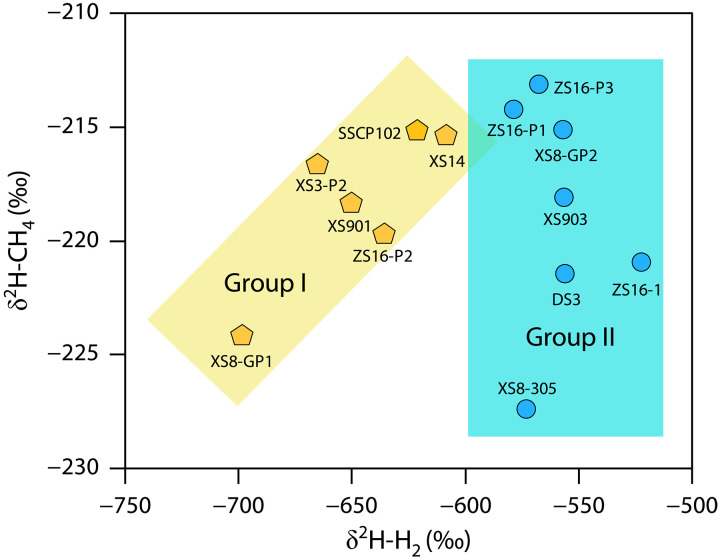
Distribution characteristics of δ^2^H-C_1_ and δ^2^H-H_2_ values in Qingshen gas deposit, Songliao Basin. The distribution characteristics of the two groups of gases are shown: one with a positive correlation between δ^2^H-C_1_ and δ^2^H-H_2_ values, and another where δ^2^H-H_2_ values are greater, while δ^2^H-C_1_ values are more dispersed. The two group gases are automatically separated at a δ^2^H-H_2_ value of −600‰.

The natural hydrogen extracted from gas samples shows a wide range of hydrogen isotope compositions (δ^2^H), from −698 to −522‰, with an average of −599‰, which is significantly heavier compared to the δ^2^H-H_2_ values in sedimentary basins worldwide (e.g., Mali, Kansas, Witwatersrand Basin) (*7*, *17*, *29*). The δ^2^H-C_1_ versus δ^2^H-H_2_ cross-plot (Fig. 3) reveals two group gases with distinct data distributions: one with a positive correlation between δ^2^H-C_1_ values and δ^2^H-H_2_ values, and another where δ^2^H-H_2_ values are greater, while δ^2^H-C_1_ values are more dispersed. These two group gases are automatically separated at a δ^2^H-H_2_ value of −600‰, which is close to the −650‰ threshold proposed in (*13*) for distinguishing between mantle-derived and crust-derived hydrogen (Fig. 3). The reason for the difference in data distribution between the two groups gases may be that the hydrogen is dominated by different origins, as discussed in the following section.

The Qingshen gas deposit of the Songliao Basin has R/Ra values (where R is the ratio of ^3^He/^4^He and Ra is the atmospheric ^3^He/^4^He ratio, approximately 1.4 × 10^−6^) ranging from 0.75 to 2.15, with an average of 1.47, indicating the presence of mantle-derived fluid (*30*, *31*). The Qingshen gas deposit was calculated to have a mantle-derived helium contribution of 18.6%, based on a crust-mantle binary mixing model (fig. S2A) (*32*), indicating that mantle-derived volatile gases migrated and accumulated in the Qingshen gas deposit (*33*). Although there is no correlation between the R/Ra value and hydrogen concentration, possibly due to substantial consumption and conversion of hydrogen (fig. S2B), the geological conditions of the study area still support the accumulation of mantle-derived gases in shallow gas deposits.

## DISCUSSION

### Origin of natural hydrogen

More than 30 pathways for the natural hydrogen origin have been identified, which can be broadly categorized into organic and inorganic origins (*11*). Organic hydrogen may be derived from the thermal evolution of sedimentary organic matter and microbial activity. The inorganic genesis of hydrogen primarily includes water-rock interactions (WRI), water radiolysis, and deep-seated (hydrogen from the Earth’s mantle or core) (*3*).

Hydrogen isotope fractionation can reveal geochemical and biochemical processes occurring on Earth (*34*). To some extent, δ^2^H-H_2_ can indicate the origin of natural hydrogen (*13*, *35*). We compiled 148 δ^2^H-H_2_ data points from around the world, representing various origins of natural hydrogen (fig. S3). The δ^2^H-H_2_ distribution range for WRI is broad, spanning −836 to −605‰. The average δ^2^H-H_2_ for water radiolysis is approximately −689‰. In the study on the origin of hydrogen in the Kansas Basin, USA, Liu *et al.* (*12*) pointed out that the δ^2^H-H_2_ values from WRI are lower than those from water radiolysis and that the hydrogen content associated with WRI is relatively higher. In contrast, δ^2^H values of mantle-derived hydrogen are significantly greater, ranging between −665 and −332‰. Organic sources and biogenic δ^2^H-H_2_ are generally lighter, ranging from −810 to −629‰ and −828 to −699‰, respectively. Mantle-derived δ^2^H-H_2_ values are notably greater than hydrogen from crustal sources, as suggested in (*13*). The wide range of δ^2^H-H_2_ values obtained in this study, with overlaps among different origins (excluding biogenesis), suggests complex origins for the natural hydrogen. Because the temperature of the Qingshen gas deposit exceeds the upper thermal barrier for life (80° to 120°C) (*36*, *37*) and the δ^2^H-H_2_ values scarcely intersect with those typical of microbial genesis, it is reasonable to dismiss biogenesis as a source of hydrogen.

The equilibrium temperatures of hydrogen isotope fractionation and the kinetics of hydrogen exchange between H_2_ and H_2_O (D-H) (kinetic isotope effects) offer additional explanations for the variations in δ^2^H-H_2_ (*38*, *39*). The hydrogen isotope thermometer provides insights into the thermal history and conditions under which geological processes occur, particularly involving H_2_O and hydrogen-containing minerals. The fractionation coefficient at hydrogen isotope equilibrium is highly sensitive to temperature (*40*). Proskurowski *et al.* (*41*) evaluated hydrogen isotope measurements of H_2_, CH_4_, and H_2_O to identify the equilibrium fractionation factor, Δ, using the standard convention

$${∆}_{A-B}=1000\text{ln}\alpha_{A-B}$$ (1)

where α_A-B_ = *R*_A_/*R*_B_, for any isotope ratio *R* (^13^C/^12^C, D/H, etc.). It should be noted that defined this way, a positive Δ_A–B_ value indicates species B is isotopically depleted with respect to species A.

**Fig. 4.**
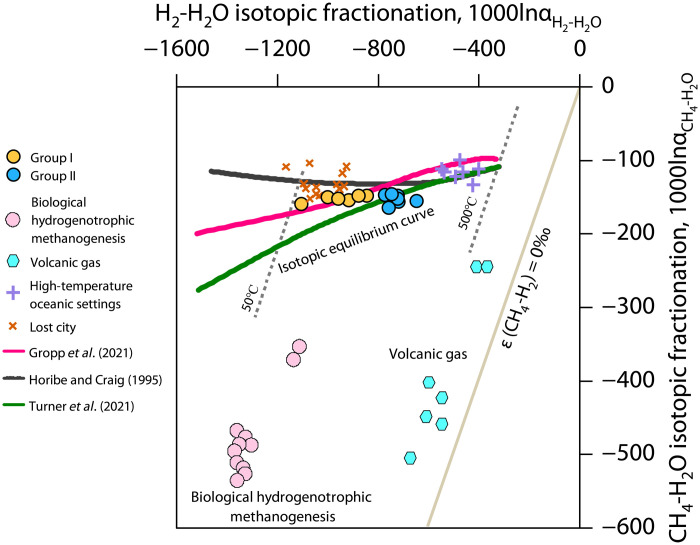
CH_4_-H_2_-H_2_O hydrogen isotope systematics: correlation between observed $1000{\mathrm{ln\alpha}}_{H_{2}-H_{2}O}$ and $1000{\mathrm{ln\alpha}}_{{\text{CH}}_{4}-H_{2}O}$ values. Hydrogen isotopic fractionation diagram modified from (*77*, *78*).

The hydrogen isotope equilibrium temperature for group I gases is relatively low, ranging from 61°C to 139°C, with an average of 105°C (Fig. 4). Conversely, the equilibrium temperature for group II gases is relatively high, ranging from 168° to 235°C, with an average of 192°C (Fig. 4). The average temperature of the Yingcheng Formation is approximately 140°C (fig. S4). Comparatively, the equilibrium temperature of group I gases is lower than the reservoir temperature, while the equilibrium temperature of group II gases is higher. This discrepancy suggests that the two groups of natural hydrogen are generated through different mechanisms at varying geological environments (temperatures or depths).

**Fig. 5.**
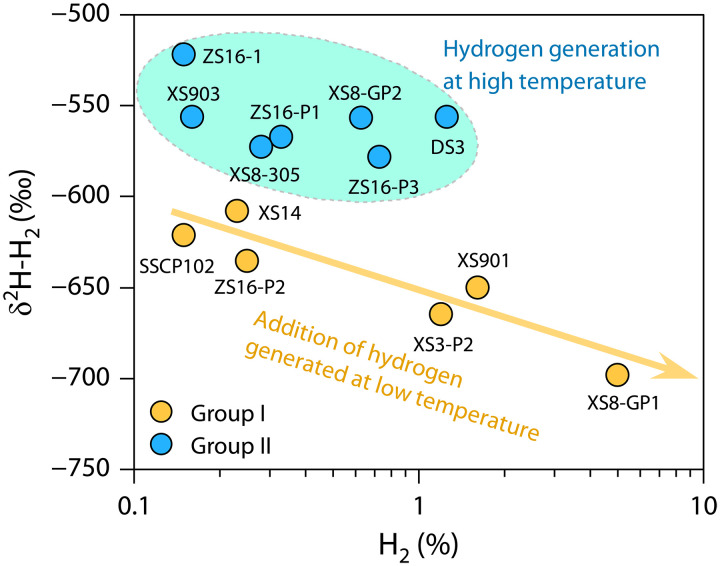
Correlation of hydrogen concentration with δ^2^H-H_2_ values for the Qingshen gas deposit, Songliao Basin.

The hydrogen concentration and δ^2^H-H_2_ values of group I gases show a strong correlation (Fig. 5). As hydrogen concentration increases, δ^2^H-H_2_ values decrease. In addition, the equilibrium temperature of hydrogen isotopes in group I gases is relatively low, indicating that this group of hydrogen may primarily be associated with generation mechanisms under low-temperature conditions within sedimentary basins, such as WRI and water radiolysis. The gradual charging of hydrogen with lower isotope values, generated under low-temperature conditions, into natural gas reservoirs results in a negative correlation between hydrogen concentration and δ^2^H-H_2_ values. The extensive distribution of volcanic rocks (e.g., olivine basalts) and mafic-ultramafic intrusions in the study area provides conditions for WRI under low temperatures (*42*). Furthermore, two high-radiation sedimentary layers were identified in the SK2 well at depths of 3096.8 to 3102.8 m and 3168.3 to 3170.0 m, with U contents ranging from 5.9 to 29.3 ppm, Th contents from 5.5 to 37.3 ppm, and K contents from 2.9 to 4.3% (*43*, *44*). Natural hydrogen generated by water radiolysis could also migrate into natural gas reservoirs.

The high-temperature environment indicated by hydrogen isotopes in group II gases aligns with the basement temperature. The SK2 well encountered high hydrogen concentrations in the basement, with an average content of 18.60%, which is significantly higher than the levels found within the basin. Under the microscope, it was found that most of the rock slices of the basement were affected by WRI (hydrothermal alteration), and Fe(II)-containing minerals (such as pyroxene and amphibole) were once developed (*43*). The hydrogen production rates during alteration of peralkaline granites at 280° to 400°C are comparable and even faster than those documented for serpentinization of olivine and harzburgite (*45*). The hydrothermal alteration of pyroxene and amphibole in the basement rocks may be the main factor for the increase of its hydrogen content, which shows part of the crustal origin.

A review of natural hydrogen by Zgonnik (*11*) summarized some direct and indirect arguments for a deep-seated origin for hydrogen, including the superdeep drilling project, metal inclusions, first natural hydride (VH_2_), and the association of hydrogen with fault zones. WRI involving mafic and ultramafic rocks in the upper mantle, the serpentinization of peridotite in subducting slabs, and primordial hydrogen support the generation of mantle-derived hydrogen (*11*, *46*–*48*). Mechanisms such as magmatic activity, mantle plumes, and deep fault channels transport mantle volatiles to the Earth’s shallow crust (*49*, *50*). Volcanic-bearing sedimentary basins, such as the Songliao Basin, located at the leading edge of a subducting plate, exhibit frequent deep fluid activity, indicating that mantle-derived hydrogen may also be one of the hydrogen sources. The magmatic gases from the Satsuma-Iwojima volcano in the eastern Pacific, characterized by typical mantle signatures (R/Ra = 7.7, CO_2_/^3^He = 8.16 × 10^8^, δ^13^C-CO_2_ = −5.3‰), allow us to use the H_2_/^3^He value (9.71 × 10^8^) from these volatiles to identify mantle-derived hydrogen (*51*). The H_2_/^3^He-R/Ra relationship diagram can distinguish between mantle-derived hydrogen and crustal-derived hydrogen (fig. S5) (*43*, *52*, *53*). Group II gases have an average R/Ra of 1.70 and an H_2_/^3^He ratio of 1.87 × 10^7^, while group I gases have an average R/Ra of 1.45 and an H_2_/^3^He ratio of 2.96 × 10^7^, indicating that both group I and group II gases are generally influenced by mantle-derived hydrogen, with group II gases containing a higher proportion of mantle-derived hydrogen. In addition, the Songliao Basin is located in a zone of multiple plate subductions and convergences, where notable lithospheric thinning and asthenospheric upwelling facilitate the entry of mantle-derived volatile gases (CO_2_, H_2_, He, etc.) into the basin (fig. S6) (*54*). The wells producing group II gases are closer to the Xuzhong deep fault compared to those producing group I gases, suggesting that mantle-derived volatiles containing hydrogen have migrated along the fault into the gas reservoirs. Figure S7 shows that high CO_2_ concentrations and a portion of helium (mean, 18.6%) originate from the mantle, demonstrating that deep fluid activity facilitates the accumulation of various abiogenic gases in the shallow sedimentary basin. Therefore, it is speculated that group II gases are related to the process in which hydrogen, generated by hydrothermal alteration of basement rocks under high-temperature conditions and from the mantle, migrates through deep faults to the Yingcheng Formation.

Horsfield *et al.* (*55*) described the generation process of organic molecular hydrogen in the Songliao Basin (fig. S8). However, the temperature and maturity criteria for hydrocarbon source rocks to generate hydrogen are high, and only some areas’ source rocks (Shahezi Formation) meet these conditions (fig. S9). Moreover, hydrogen generated during the maturation of kerogen is likely consumed by the aforementioned hydrogenation reactions. This is supported by the low hydrogen yields from pyrolysis of source rocks in both open and closed systems (*56*). It should be noted that we do not exclude the presence of organic hydrogen, as reported in (*55*), but the proportion of organic hydrogen in natural gas reservoirs may not be substantial.

Under experimental conditions, it was found that water directly participates in the generation of natural gas (*57*). Formation water engages in both low-temperature WRI and hydrogen exchange with hydrocarbon source rocks, thereby affecting the δ^2^H values of both hydrogen and alkane gases. This explains the positive correlation exhibited by the group I gases in Fig. 3. The hydrogen in the group II gases primarily originates from high-temperature WRI occurring in basement rocks and from the mantle. Since the water in the basement does not interact with hydrocarbon source rocks, no correlation is observed between the δ^2^H values of CH_4_ and hydrogen in the group II gases in Fig. 3.

**Fig. 6.**
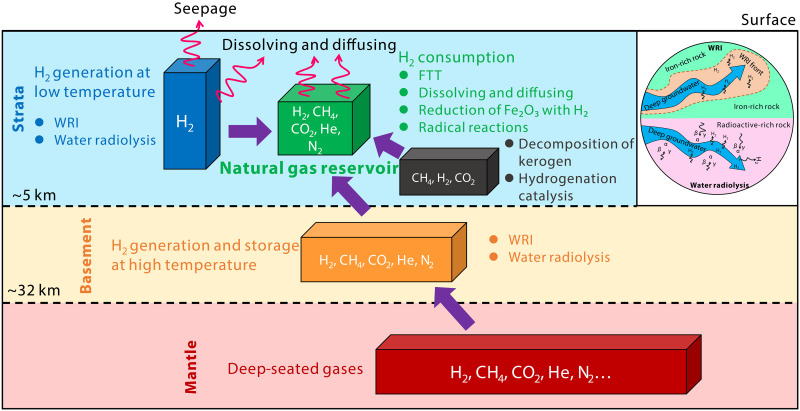
The crust-mantle mixed origin system of natural hydrogen in the volcanic-bearing sedimentary basin.

Here, we have established a crust-mantle mixed origin system for natural hydrogen in the volcanic-bearing sedimentary basin (Fig. 6). Specifically, natural hydrogen of the Qingshen gas deposit originates from low-temperature WRI in iron-rich rocks and water radiolysis of radioactive elements of source rocks within the basin, high-temperature WRI (hydrothermal alteration) and water radiolysis in basement rocks, as well as deep-seated hydrogen. Hydrogen in natural gas reservoirs may be consumed through Fischer-Tropsch–type (FTT) reactions, dissolution, diffusion, reduction of iron-containing minerals (*58*), and radical reactions between hydrogen and heavier gaseous hydrocarbons (*59*).

### Conversion and original resource of natural hydrogen

The origin of alkane gases, especially in the Qingshen gas deposit of the Songliao Basin, has always been a controversial issue. The presence of heavier δ^13^C_1_ values and the inverse feature in alkane carbon isotopes have been suggested as indicators of abiotic origins of alkane gases (*60*). According to Whiticar’s carbon and hydrogen isotope scheme for the identification of the origin of CH_4_ (*28*), the isotope values for the Qingshen gas deposit indicate that CH_4_ formed under geothermal, hydrothermal, and crystalline conditions (fig. S10). In areas like the Songliao Basin, where there is deep fluid activity and faulted basins contain certain concentrations of hydrogen and mantle-derived CO_2_, we must consider the contribution of abiotic CH_4_ to natural gas reservoirs, including FTT reactions and mantle-derived CH_4_ (*19*, *61*–*63*).

**Fig. 7.**
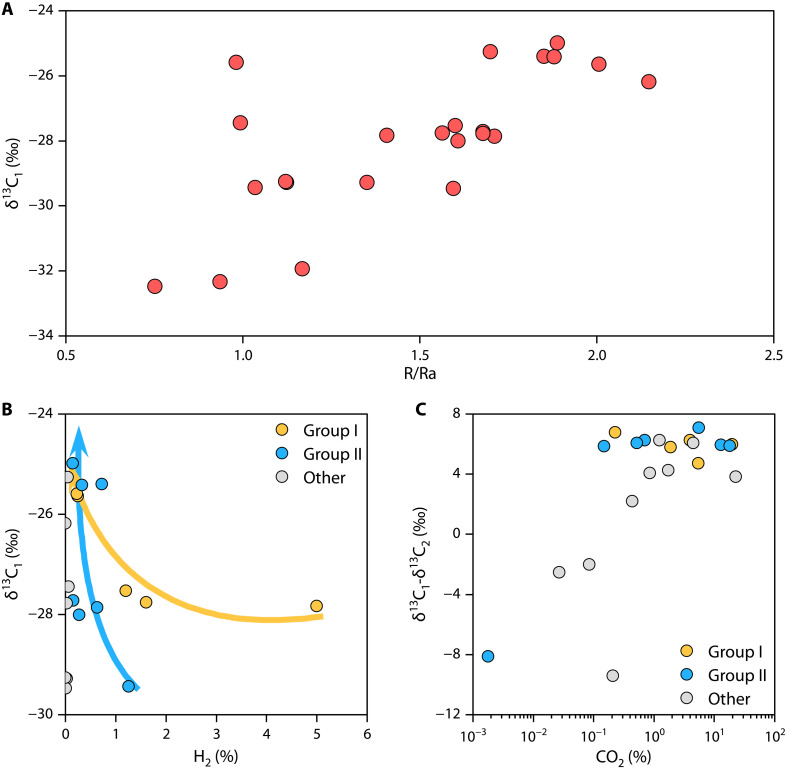
Contribution of abiotic CH_4_ to natural gas reservoirs. (**A**) There is a positive correlation between R/Ra and δ^13^C_1_ values. (**B**) As hydrogen content decreases, δ^13^C_1_ values become greater. (**C**) Relationship between CO_2_ content and δ^13^C_1_-δ^13^C_2_ values.

Figure 7A shows that as R/Ra increases, δ^13^C_1_ tends to become greater, indicating the presence of mantle-derived abiotic CH_4_. As hydrogen content decreases, δ^13^C_1_ increases (Fig. 7B), suggesting increased abiotic gas components. This change reflects the FTT reactions, where hydrogen reacts with CO_2_ to form abiotic CH_4_. When residual CO_2_ content is high, the δ^13^C_1_ and δ^13^C_2_ typically exhibit reversal (Fig. 7C), indicating that the FTT reactions partially consume a large amount of mantle-derived CO_2_ until hydrogen content is insufficient to sustain it, leaving excess CO_2_. Conversely, samples with low CO_2_ content may not be influenced by mantle degassing, resulting in an insufficient carbon source for the FTT reactions, and thus no reversal in alkane carbon isotopes. R/Ra, CO_2_/^3^He, and CH_4_/^3^He ratios are used to infer the possible crustal or mantle origins of gases (*64*). Almost all samples fall into the lower part of the magmatic range, and relative to ^3^He, they appear to have experienced CO_2_ loss and dissolution (fig. S11A). As shown in fig. S11B, most of the gas samples from the Songliao Basin are above the two-component mixing area between crustal and mantle endmembers, suggesting that additional CH_4_ input has resulted in high CH_4_/^3^He values in gas samples associated with high R/Ra ratios. The CH_4_/^3^He values in the other group gases, where δ^2^H-H_2_ could not be measured due to low hydrogen concentrations, are higher than in groups I and II. This reveals that a substantial amount of hydrogen has been consumed and converted into additional CH_4_. The loss of CO_2_ and the anomalously high levels of CH_4_ are indicative of the FTT reactions.

The geological setting characterized by deep fluid activity facilitates the conversion of hydrogen from various origins and mantle-derived CO_2_ into CH_4_, yielding δ^13^C_1_ signatures (*65*). The efficiency of FTT reactions is contingent on both the presence of catalysts and specific thermal conditions. Iron, nickel, cobalt, chromium, and minerals containing these metals are recognized catalysts in FTT reactions, enabling the reaction to occur naturally within geological formations even under conditions of low temperature and pressure, albeit over extended periods (*18*, *63*). For example, natural gas from serpentinized peridotites in Chimaera, containing chromite, with 9.76% H_2_ and 85.7% CH_4_, is believed to have been generated through low-temperature (<50°C) FTT methanation (*6*). The Yingcheng Formation and the basin’s basement, both rich in iron-containing mafic rocks, are potential sites for the FTT reactions to produce abiotic CH_4_. This CH_4_ then migrates and mixes with organically derived gases to form natural gas reservoirs.

The linear mixing model proposed in (*64*) was used to estimate the CH_4_ content from different origins (Supplementary Materials). The results show that the proportion of abiotic CH_4_ in the Qingshen gas deposit ranges from 24.99 to 49.26% (excluding wells DS3 and DS17), with an average of 37.75%. This result is consistent with the distribution characteristics of δ^13^C values of the Qingshen gas deposit on the mixing lines between thermogenic and abiotic endmembers established in (*6*) (fig. S12). By 2018, the proven natural gas reserves of the Qingshen gas deposit had reached 252.271 × 10^9^ m^3^ (*64*). On the basis of previous calculations, it is estimated that the Qingshen gas deposit contains approximately 95.23 × 10^9^ m^3^ of abiotic CH_4_. Therefore, the abiotic alkane gas in the Qingshen gas deposit has promising exploration prospects. Abiotic alkane gas can form commercial accumulations under specific geological conditions, which holds positive and practical significance for expanding natural gas exploration in sedimentary basins.

**Fig. 8.**
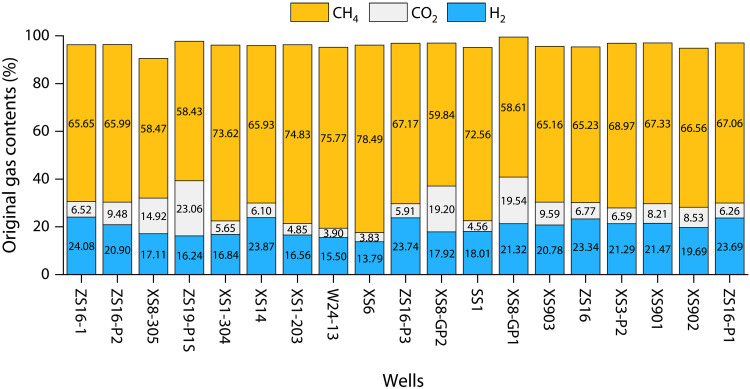
Maximum original gas contents in the Qingshen gas deposit.

A mixing model using crust-mantle endmembers (with δ^13^C_1_ values set at −30.0 and −15.0‰, respectively) calculates that the proportions of mantle-derived and FTT CH_4_ in natural gas are 31.70 and 6.04%, respectively. Approximately 15.24 × 10^9^ m^3^ of CH_4_ is estimated to be derived from FTT reactions. The FTT reactions between H_2_ and CO_2_, which produce 1 mol of CH_4_, require the consumption of 4 mol of H_2_ and 1 mol of CO_2_. Using the gas conversion ratios from the FTT reactions, we estimated the proportion of CO_2_ and H_2_ involved in the conversion. The FTT reactions consumed up to 15.24 × 10^9^ m^3^ of mantle-derived CO_2_. Using the average CO_2_ content of 4.4% in the Qingshen gas deposit, the current CO_2_ reserves are calculated to be around 3.79 × 10^9^ m^3^. This indicates that at least 19.03 × 10^9^ m^3^ of mantle-derived CO_2_ was released into the shallow crust. After accounting for the conversion, we calculated the maximum original hydrogen concentrations in the gas reservoir range from 13.79 to 24.08%, with an average of 19.80% (Fig. 8). Further estimates suggest that the maximum original hydrogen reserves in the Qingshen gas deposit are 61.9 × 10^9^ m^3^. Here, we state that the calculated original hydrogen contents are overestimated (representing the maximum original hydrogen content), as FTT reactions also occurs outside the reservoir (e.g., in the basin basement), where the natural hydrogen within the reservoir is not consumed.

With an average hydrogen concentration of 0.53% in the collected gas samples, the current hydrogen reserves in the Qingshen gas deposit are estimated to be 1.34 × 10^9^ m^3^. The results indicate that approximately 97.8% of hydrogen has been consumed through conversion. Natural gas systems in volcanic-bearing sedimentary basins can serve as sites for the accumulation of large amounts of natural hydrogen. However, hydrogen from different origins is often substantially consumed, leading to generally low hydrogen concentrations in current natural gas reservoirs.

### Production rates of natural hydrogen

Overall, natural hydrogen in the Songliao Basin primarily originates from water radiolysis, WRI, and mantle. Cheng *et al.* (*66*) investigated the proportion of gas loss due to dissolution and diffusion during the migration of degassed gases from the deep crust to the overlying strata. In contrast, this paper primarily assesses the natural gas system’s capacity to trap hydrogen from different origins. We assume that natural gas reservoirs can only store hydrogen generated beneath them. In addition, we developed a geological model of the source-reservoir combination for natural hydrogen in the volcanic-bearing sedimentary basins to facilitate the calculation of hydrogen store rates. This model includes several layers, ranging from shallow to deep: natural gas reservoirs (traps), strata (extending from the natural gas reservoirs to the top of the basement), part of the upper crust (from the top of the basement to the top of the middle crust), the middle crust, the lower crust, and the mantle (mantle-derived hydrogen). We did not consider the influence of hydrogen generation in the middle and lower crust, as their water-filled porosity may be insufficient to support hydrogen production. The basic geological parameters of this model can be found in the Supplementary Materials.

The natural emission of α, β, and γ particles resulting from the decay of uranium (U), thorium (Th), and potassium (K) was assessed in (*67*, *68*) for typical granite, basalt, and quartzite lithologies. This assessment served as the foundation for calculating radiolytic hydrogen production. In the radiolytic hydrogen model, Sherwood Lollar *et al.* (*5*) derived a crust fracture porosity related radiolytic hydrogen rate of 16 × 10^9^ mol H_2_ year^−1^(358.0 MMm^3^/year at standard temperature and pressure) for global Precambrian continental lithosphere. On the basis of the genetic method in (*5*, *14*), we calculated a maximum radiolytic hydrogen production rate of 286.0 MMm^3^/year and a ^4^He production rate of 5.9 MMm^3^/year in the Songliao Basin. The earliest time of natural gas accumulation is approximately 100 Ma (*69*), which may also be when natural hydrogen began to be trapped. The Songliao Basin has generated a maximum total of 28.6 × 10^12^ m^3^ of radiolytic hydrogen over this time. Further calculations indicate that the crust below the Qingshen gas deposit in the Xujiaweizi Depression has generated up to 572 × 10^9^ m^3^ of radiolytic hydrogen.

Several studies have provided estimates of global hydrogen production from marine systems, including both volcanic/magmatic sources and hydrogen production from the abiogenic WRI that are the focus of this paper (*5*). Water radiolysis based on radioelement concentration and porosity is tied to ^4^He production, and mineral hydration dominantly associated with rock type and independent of noble gas production (*22*). Most studies follow the approach typical for the marine literature, using reaction-based models with a governing equation relating oxidation of FeO in the crust to hydrogen production at a ratio of 3:1 that is either of the form of equation 2 of (*70*).

$$\text{3FeO}\;\left(\text{in silicates}\right)+H_{2}O\to {\text{Fe}}_{3}O_{4}\;\left(\text{magnetite}\right)+H_{2}$$ (2)

In our study, we used the numerical method of Sherwood Lollar *et al.* (*5*) to quantify the hydrogen produced through WRI. Briefly, the 2014 model estimated that every 1 m^3^ of near-surface mafic and ultramafic rock had the potential to produce up to 1400 mol of H_2_ through WRI. If the efficiency of WRI reaches 100%, the calculations indicate that WRI in rocks from the depth of the Qingshen gas deposit in the Songliao Basin (3 km) to the bottom of the upper crust (12.75 km) has produced a total of 1.16 × 10^18^ mol of H_2_. On the basis of the age of the strata [113 Ma; (*71*)] and basement rocks [954 Ma; (*72*)], a hydrogen production rate of 5.53 × 10^9^ mol/year from WRI was estimated, equivalent to 124.0 MMm^3^ H_2_ year^−1^. The cumulative maximum production of WRI hydrogen in the Xujiaweizi Depression since 100 Ma was calculated to be 248 × 10^9^ m^3^.

The flux of mantle-derived hydrogen in natural gas reservoirs was estimated using mantle-derived hydrogen and helium from the magmatic volatiles of the Satsuma-Iwojima volcano in the eastern Pacific. According to (*51*), the maximum H_2_/^3^He value in these volatiles is approximately 9.71 × 10^8^. The average ^3^He content in the Qingshen gas deposit is 5.19 × 10^−8^. Using the maximum H_2_/^3^He value from the Satsuma-Iwojima volcano, the maximum total production of mantle-derived hydrogen is estimated to account for 50% of the Qingshen gas deposit’s reserves, amounting to approximately 127 × 10^9^ m^3^.

On the basis of the hydrogen production rates from radiolysis, WRI, and mantle sources, a maximum total of 947 × 10^9^ m^3^ of natural hydrogen has been generated in the lower part of the Qingshen gas deposit. The hydrogen store rate of the restored natural gas system is 6.6%, while the current natural gas system (after consumption and conversion) has a hydrogen store rate of 1.4% (table S2). These data indicate that the hydrogen store rate of natural gas systems can be considerable, particularly in volcanic-bearing sedimentary basins. Given the favorable geological conditions for trapping natural hydrogen and the substantial gas reserves in natural gas deposits, the hydrogen within natural gas systems deserves considerable attention.

### The formation model of natural hydrogen in the Songliao Basin

Unlike other geological structures, the formation process of hydrogen-rich natural gas reservoirs in volcanic-bearing sedimentary basins is more complex. This process is analogous to the generation of oil and gas from source rocks and their subsequent migration to traps for accumulation. However, it often involves multiple hydrogen-generating geological processes and interactions between different spheres of the Earth.

**Fig. 9.**
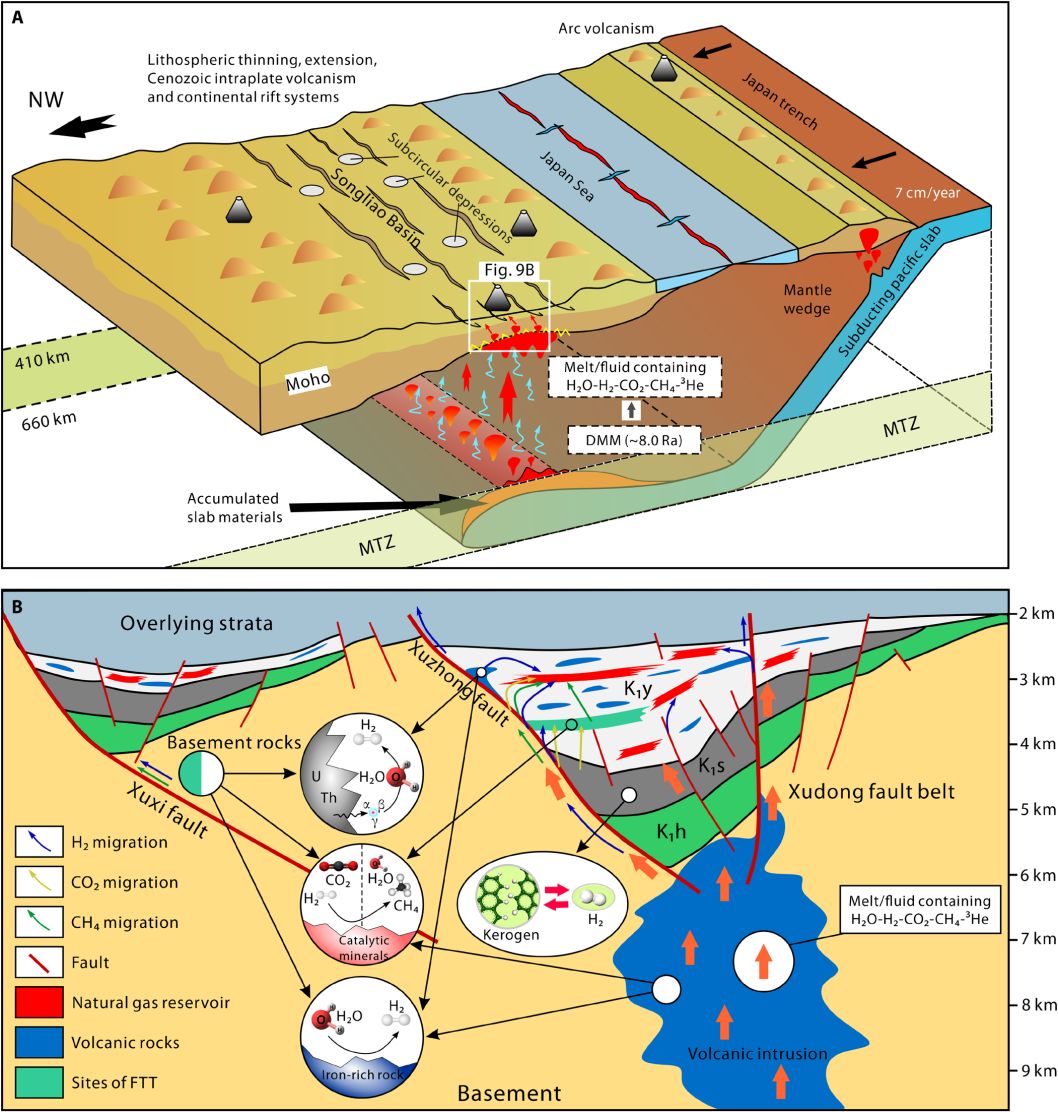
The formation model of natural hydrogen in the Songliao Basin. (**A**) Under the subduction of the Paleo-Pacific Plate, deep fluids carrying volatiles traverse lithospheric layers into the shallow crust and release gases, modified from (*79*). NW, northwest; MTZ, mantle transition zone; DMM, depleted upper mantle. (**B**) Sedimentary basins and basement rocks generate and consume hydrogen through WRI, water radiolysis, decomposition of organic matter, and FTT reactions. The geological structural framework of the basin is modified from (*80*), and the kerogen structure is sourced from (*81*).

In the Songliao Basin, there is intense interaction between the shallow crust and the deep mantle. Deep fluids, as important carriers of materials and energy from the Earth’s interior, facilitate the transfer of matter and energy across Earth’s spheres through major structural channels. This phenomenon is common in active tectonic zones such as continental rift basins and mid-ocean ridges. Volatile gases from the mantle, as essential components of Earth’s deep fluids, accompany magmatic effusion and intrusion activities during mantle degassing. These gases mainly include CO_2_, H_2_O, SO_2_, H_2_S, N_2_, CH_4_, H_2_, and rare gases (*73*, *74*). Upwelling of asthenospheric material beneath the Songliao Basin, along with partial melting and thinning of the crust, reduces the stress exerted by the overlying rocks, creating favorable conditions for the ascent of deep fluids. Deep fluids carrying mantle-derived volatile gases disperse upward along trans-crustal faults and are trapped by geological bodies capable of storing hydrogen, forming natural hydrogen gas accumulations (Fig. 9A).

The basement of the Songliao Basin is a notable site for hydrogen generation. WRI (iron-rich rocks and water), radiolytic decomposition of water, and injection of mantle-derived volatiles are reasons for the high concentrations of hydrogen in the basement. Natural hydrogen in the basement can migrate through fault systems into the Songliao Basin and be stored by hydrogen-storing geological bodies, forming hydrogen gas accumulations (Fig. 9B) (*50*). The sedimentary strata of the Songliao Basin also have the capacity to generate hydrogen. Organic matter in the Shahezi Formation, under high temperatures and a high degree of thermal evolution, produces organically natural hydrogen (*55*), which may then enter natural gas reservoirs through oil and gas migration pathways. The mafic intrusive rock bodies within the basin can generate hydrogen through WRI at low temperatures. In addition, the decay of radioactive elements in volcanic rocks can decompose water to produce hydrogen. Besides diffusion loss, the FTT reaction occurring in the basement and sedimentary strata is another important mechanism for hydrogen loss. Mixing of varying concentrations of abiotic and biotic CH_4_ may occur in the deep natural gas reservoirs of the Songliao Basin.

## MATERIALS AND METHODS

### Sampling

Natural gas was meticulously collected from the Yingcheng Formation of the Qingshen gas deposit across 23 wells within the Xujiahuizi fault depression, Songliao Basin. The collected gas samples were ensured to be pure by directly extracting them from wellheads within operational hydrocarbon deposits. A preliminary step involved purging the lines for a duration of 15 to 20 min, a process aimed at eliminating any potential air impurities, thereby ensuring the integrity of the samples for subsequent analysis. We used a robust stainless steel cylinder, precisely 25 cm in diameter and capable of holding about 10 liters of gas. These cylinders were fitted with dual shut-off valves designed to withstand up to 22.5 MPa. The protocol required maintaining internal pressure above 5.0 MPa to avoid air contamination, with a thorough water immersion leak test following sample collection.

### Natural gas composition analysis

Analyses for determining the relative molecular composition of the gases were performed by gas chromatograph in the laboratory of the Oil and Gas Resources Research Center, Northwest Institute of Eco-Environment and Resources, Chinese Academy of Sciences. Hydrocarbon gases were analyzed on a GC5890N equipped with a flame ionization detector and an Al_2_O_3_ column (30 m by 0.53 mm), with N_2_ as the carrier gas. The oven temperature program for the Al_2_O_3_ column was set to 0°C for 3 min, followed by heating at 10°C/min to 180°C (5 min hold). Nonhydrocarbon gases (such as H_2_, N_2_, and He) were analyzed using a GC9790 equipped with a thermal conductivity detector and a TDX-01 column (3 m by 3 mm). The oven temperature program for the TDX-01 column started at 40°C for 5 min, followed by heating at 10°C/min to 240°C (10-min hold).

### Isotope analysis

Stable carbon isotope compositions were accurately measured using a Finnigan MAT-253 instrument. This detailed gas chromatographic analysis involved a Porapak Q column and carefully controlled temperature settings to progressively increase from 40° to 160°C. Pure helium was used as the carrier gas at a flow rate of 1.2 ml/min, with the analytical precision for δ^13^C values maintained within <0.3‰. Each sample was measured three times, and the results of the three measurements were averaged. δ^2^H measurements were conducted on a Deltaplus XP mass spectrometer under finely tuned gas chromatography conditions, ensuring the precision of δ^2^H values to within 3.0‰, Vienna standard mean ocean water. The minimum hydrogen concentration for δ^2^H measurements is 0.1%. Each sample was measured three times, and the results of the three measurements were averaged. The ^3^He/^4^He ratios were determined with a Noblesse noble gas mass spectrometer (Nu Instruments, UK) calibrated with air from the Gaolan Hill area south of Lanzhou.

## Supplementary Materials

### This PDF file includes:

Supplementary Text Figs. S1 to S12 Tables S1 and S2 References
