# Supplementary material for: Natural hydrogen in the volcanic-bearing sedimentary basin: Origin, conversion, and production rates
Source: Sci Adv. 2025 Jan 24;11(4):eadr6771. doi: 10.1126/sciadv.adr6771 (PMC11759049; doi:10.1126/sciadv.adr6771)
Supplement: Supplementary file 1 — Supplementary Text Figs. S1 to S12 Tables S1 and S2 References [file sciadv.adr6771_sm.pdf]

Science Advances
AAAS

Supplementary Materials for

Natural hydrogen in the volcanic-bearing sedimentary basin: Origin, conversion, and production rates

Quanyou Liu *et al.*

Corresponding author: Quanyou Liu, liuqy@pku.edu.cn, qyouliu@sohu.com;
Yongbo Wei, weiyongbo@mail.iggcas.ac.cn

Sci. Adv. **11**, eadr6771 (2025)
DOI: 10.1126/sciadv.adr6771

This PDF file includes:

Supplementary Text
Figs. S1 to S12
Tables S1 and S2
References

Supplementary Text

Linear mixing model calculations of CH_4 from different origins

For the natural gas in the Qingshen gas deposit of the Songliao Basin, the $\delta^{13}C_1$ value of the natural gas from Well Fangshen 2 is -17.4‰. The alkane gas exhibits a typical negative carbon isotope series ($\delta^{13}C_1 > \delta^{13}C_2 > \delta^{13}C_3 > \delta^{13}C_4$) and an R/Ra value of 5.84, which is consistent with the characteristics of typical abiotic gas (*64*). Therefore, it can be considered a typical end-member of abiotic gas. The natural gas from the nearby Chaoyanggou gas deposit was analyzed for the end-member of biogenic gas. This gas deposit's typical oil-type gas and coal-derived gas have $\delta^{13}C_1$ and $\delta^{13}C_2$ values with a significant positive correlation, with the fitting formulas of $\delta^{13}C_1$= 0.8957×$\delta^{13}C_2$-5.3104 and $\delta^{13}C_1$=0.9722×$\delta^{13}C_2$-8.4681, respectively, showing the control of maturity on the carbon isotope composition (*65*). Given that the $\delta^{13}C_2$ values of typical oil-type gas and coal-derived gas are less than and greater than -28.0‰, respectively (*82*), and typical abiotic gas is primarily CH_4 with abnormally low heavy hydrocarbon content, these fitting formulas can be used to calculate the corresponding $\delta^{13}C_1$ values of the biogenic gas end-member by substituting the measured $\delta^{13}C_2$ values of the Qingshen gas deposit samples. Subsequently, the measured $\delta^{13}C_1$ values of the biogenic gas end-member and $\delta^{13}C_1$ values of the abiotic gas end-member from Well Fangshen 2, combined with the $\delta^{13}C_1$ values of the gas samples, can be used in linear mixing model calculations to determine the proportions of abiotic and biogenic CH_4 in the samples.

Abiotic CH_4 can be classified into two sources: mantle degassing and FTT reactions (*63*). At the 21°N mid-ocean ridge of the East Pacific, hydrothermal emissions contain CH_4, H_2, and He, with $\delta^{13}C_1$ values ranging from -17.6‰ to -15.0‰ and R/Ra values of approximately 8.0, indicating these gases are of typical mantle origin (*83*). Therefore, the $\delta^{13}C$ value of CH_4 from mantle degassing can be set at -15.0‰. Currently, there is no consensus on the $\delta^{13}C_1$ value of CH_4 synthesized via FTT. However, due to kinetic fractionation effects between ^{12}C and ^{13}C, CH_4 synthesized via FTT is relatively enriched in ^{12}C, and its $\delta^{13}C_1$ value should not be lower than the threshold distinguishing abiotic and biogenic CH_4 (-30.0‰) (*84*). Thus, the $\delta^{13}C$ value of CH_4 from the FTT end-member can be set at -30.0‰. For the Qingshen gas deposit in the Songliao Basin, the $\delta^{13}C$ value of abiotic alkane gas from Well Fangshen 2 is -17.4‰. Based on a mixing model using crust-mantle endmembers (with $\delta^{13}C_1$ values set at -15.0‰ and -30.0‰, respectively), the proportions of mantle-derived and FTT CH_4 are estimated to be 84% and 16%, respectively. The relevant calculation formulas are as follows:

$$\delta^{13}C_{1,\text{ biotic }CH_4}=0.8957\times\delta^{13}C_{2,\text{ sample}}-5.3104 \quad (S1)$$

$$R_{\text{abiotic }CH_4}=\frac{\left(\delta^{13}C_{1,\text{ sample}}-\delta^{13}C_{1,\text{ biotic }CH_4}\right)}{\left(-17.4-\delta^{13}C_{1,\text{ biotic }CH_4}\right)}\times 100\% \quad (S2)$$

$$R_{\text{FTT }CH_4}=R_{\text{abiotic }CH_4}\times 0.16 \quad (S3)$$

$$R_{\text{FTT }CH_4\text{ in sample}}=R_{CH_4}\times R_{\text{FTT }CH_4} \quad (S4)$$

$$L_{\text{FTT }H_2}=4\times R_{\text{FTT }CH_4\text{ in sample}} \quad (S5)$$

$$L_{\text{FTT }CO_2}=R_{\text{FTT }CH_4\text{ in sample}} \quad (S6)$$

$$M_{\text{tot }H_2}=L_{\text{FTT }H_2}+R_{H_2} \quad (S7)$$

$$M_{\text{tot }CO_2}=L_{CO_2}+R_{CO_2} \quad (S8)$$

$$M_{\text{tot gas}}=M_{\text{tot }H_2}+M_{\text{tot }CO_2}+(R_{CH_4}-R_{\text{FTT }CH_4})+(100\%-R_{H_2}-R_{CO_2}-R_{CH_4}) \quad (S9)$$

$$R_{\text{original }H_2}=\frac{M_{\text{tot }H_2}}{M_{\text{tot gas}}}\times 100\% \quad (S10)$$

where $\delta^{13}C_{1,\text{ biotic }CH_4}$ represents the carbon isotope end-member value of biotic CH_4, $\delta^{13}C_{1,\text{ sample}}$ represents the carbon isotope value of CH_4 in the gas sample, $\delta^{13}C_{2,\text{ sample}}$ represents the carbon isotope value of C_2H_6 in the gas sample, $R_{\text{abiotic }CH_4}$ is the content of abiotic CH_4, $R_{\text{FTT }CH_4}$ is the content of FTT CH_4 in CH_4, $R_{\text{FTT }CH_4\text{ in sample}}$ is the content of FTT CH_4 in gas sample, R_{CH_4} is the content of CH_4 in gas sample, $L_{\text{FTT }H_2}$ represents the hydrogen content consumed by FTT, $L_{\text{FTT }CO_2}$ represents the CO_2 content consumed by FTT, $M_{\text{tot }H_2}$ is the restored H_2 content (non-normalized), $M_{\text{tot }CO_2}$ is the restored CO_2 content (non-normalized), R_{H_2} is the content of H_2 in gas sample, R_{CO_2} is the content of CO_2 in gas sample, $M_{\text{tot gas}}$ represents the total gas content after recovery, and $R_{\text{original }H_2}$ represents the normalized maximum original content of H_2.

Basic geological parameters for calculating hydrogen production rates

For the Qingshen gas deposit, the average depth of the natural gas reservoir is 3 km. In the Songliao Basin, the average depth of the top of the basement is 6.45 km, the depth of the top of the middle crust is 12.75 km, and the depths of the top and bottom of the lower crust are 21.6 km and 34.4 km, respectively (*85*). The thicknesses of the strata, part of the upper crust, middle crust, and lower crust in the geological model are 3.45 km, 6.3 km, 8.85 km, and 12.8 km, respectively. The area of the Songliao Basin is 260×10^3 km^2, and the Xujiaweizi Depression, where the Qingshen gas deposit is located, covers an area of 5 350 km^2.

The contents of U, Th, and K in the strata of the Songliao Basin are approximately 2.57 ppm, 6.79 ppm, and 2.70%, respectively (*86*). In the basin's basement (representing part of the upper crust), the concentrations of U, Th, and K are approximately 6.00 ppm, 12.35 ppm, and 3.85%, respectively (*87*). It has been reported that the porosity of strata rocks below 3 km in sedimentary basins is approximately 2% (*87*). The density of crustal rocks is 2.656 g/cm^3, and the average porosity of the upper basement rocks in the Songliao Basin is 0.78% (*88*). To account for the variation in porosity with crustal depth, we can estimate the minimum amount of water available for radiolysis in the continental crust by assuming, based on Bethke's (*89*) model, that the porosity of water-filled fractures decreases exponentially with depth in kilometers. The calculated average porosity for part of upper crust is 0.45%. Depending on previous study, we assumed that the FeO content in the strata of volcanic-bearing sedimentary basins and part of upper crust to be 5.00% and 3.02%, respectively (*90*).

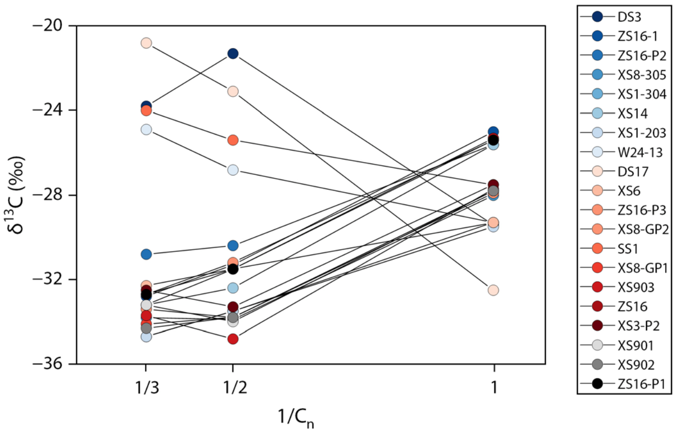

Fig. S1. The carbon isotope variation characteristics of natural gas from the Qingshen gas deposit of the Songliao Basin.

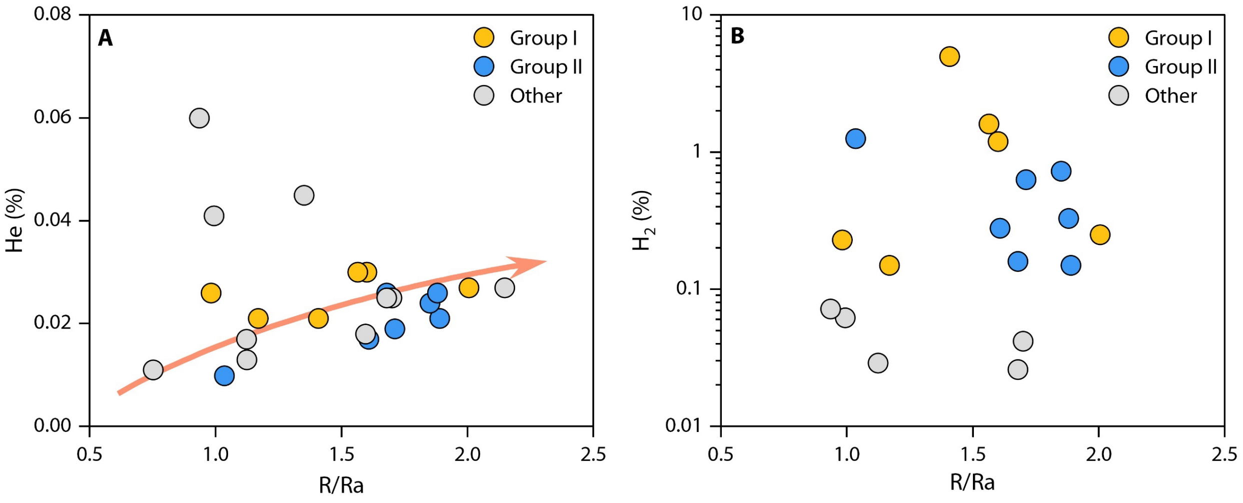

Fig. S2. Correlation of R/Ra with hydrogen (A) and helium (B) concentrations for the Qingshen gas deposit, Songliao Basin.

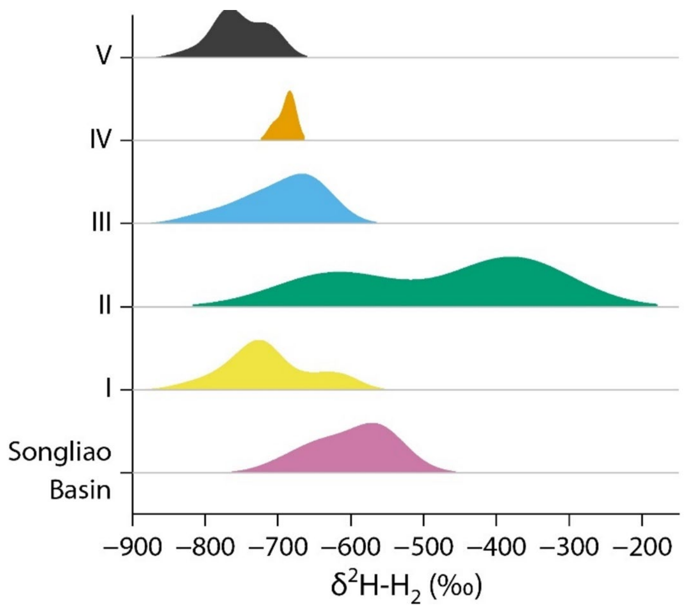

Fig. S3. Hydrogen isotope values for natural hydrogen from different origins. I: Water-rock interactions at low temperatures, particularly through serpentinization within the crust. II: Hydrogen derived from the mantle. III: Hydrogen is generated from overmature organic matter in shales or coals. IV: The decay of radioactive elements in the Earth's crust rocks releases α、β and γ rays, which produce energy that breaks down water molecules into oxygen and hydrogen. V: Hydrogen produced by microbial action. Data are from (*6-8, 9,17, 29, 41, 91-96*).

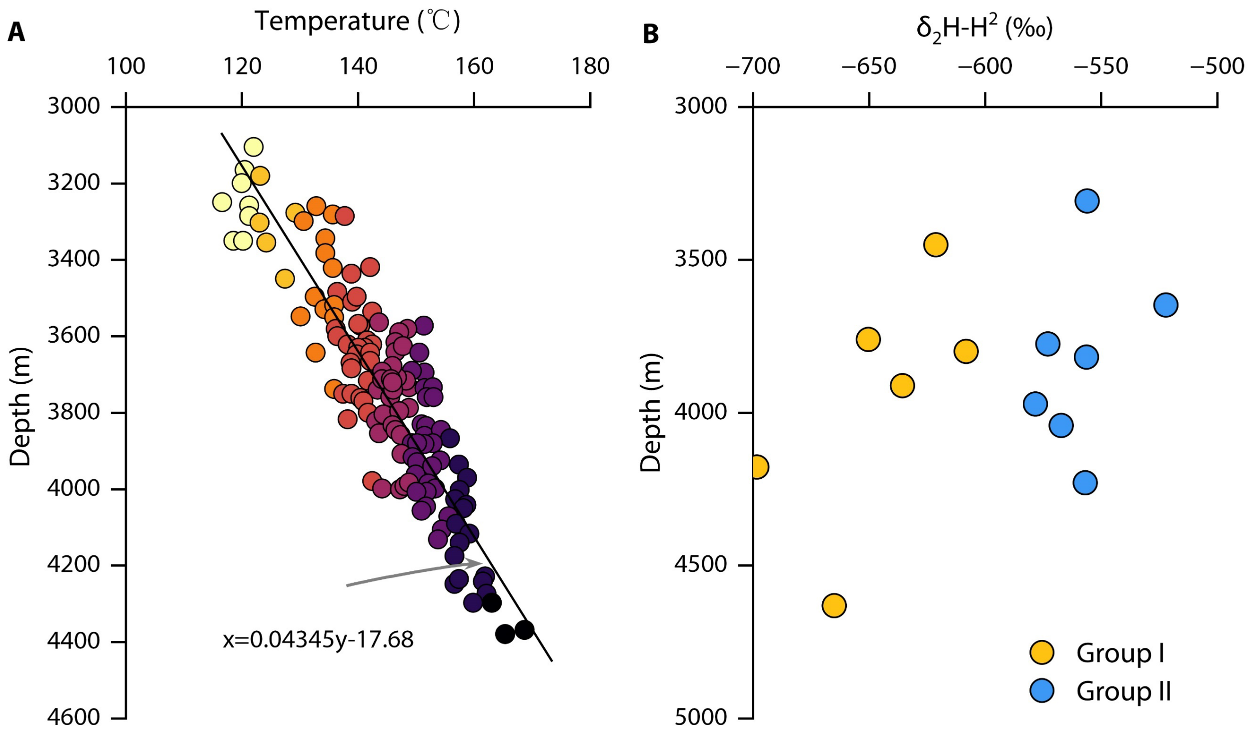

Fig. S4. The relationship between depth, temperature, and $\delta^2H\text{-}H_2$ values. (A) The relationship between temperature and depth of the Yingcheng and Huoshiling Formations in the Xujiaweizi Depression. Data are from (*97*). (B) The relationship between $\delta^2H\text{-}H_2$ values and sampling depths.

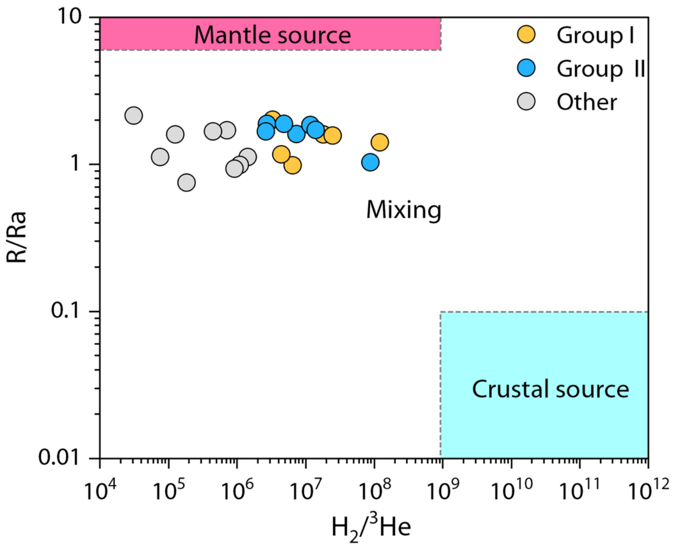

Fig. S5. Genetic identification of hydrogen by R/Ra and $H_2/^3He$ values, modified from (*43*).

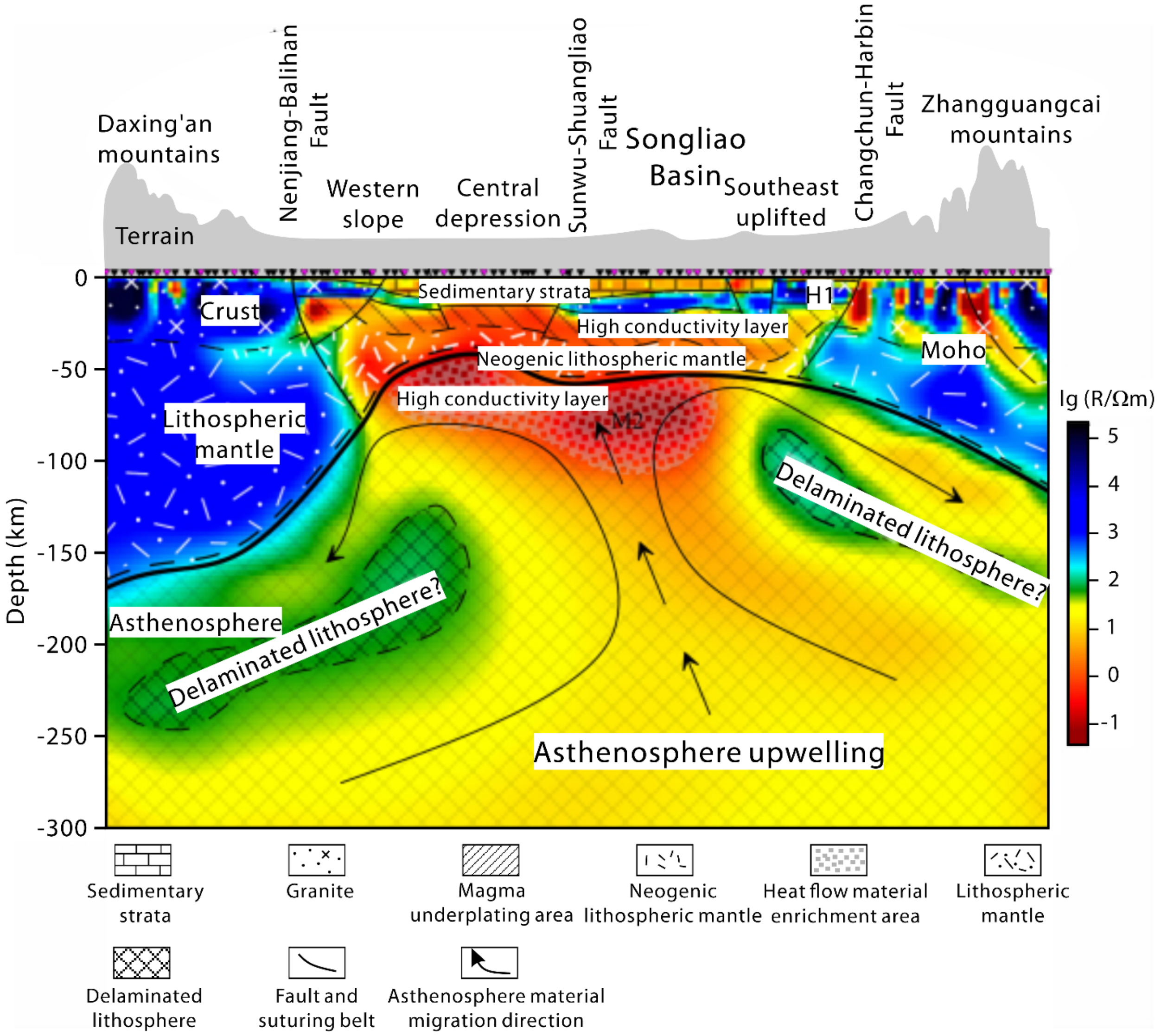

Fig. S6. Two-dimensional electrical structure of Songliao Basin and its inference, modified from (*98*).

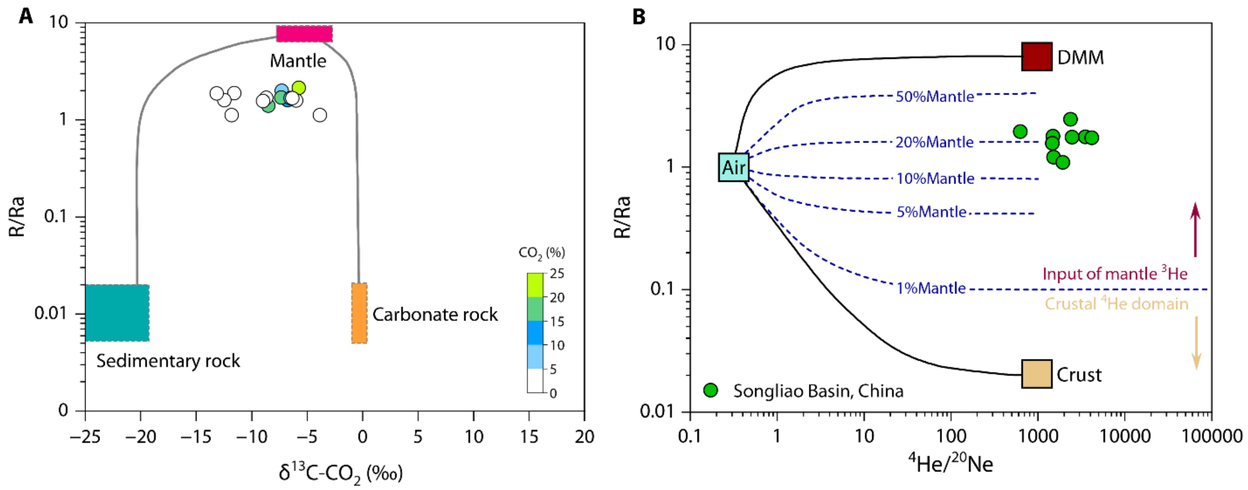

Fig. S7. Evaluation of mantle-derived gases in the Qingshen gas deposit of the Songliao Basin. (A) δ^{13}C-CO_2 vs. R/Ra, modified from (*6*). (B) ^{4}He/^{20}Ne vs. R/Ra, modified from (*99*). Data are from (*64, 100*).

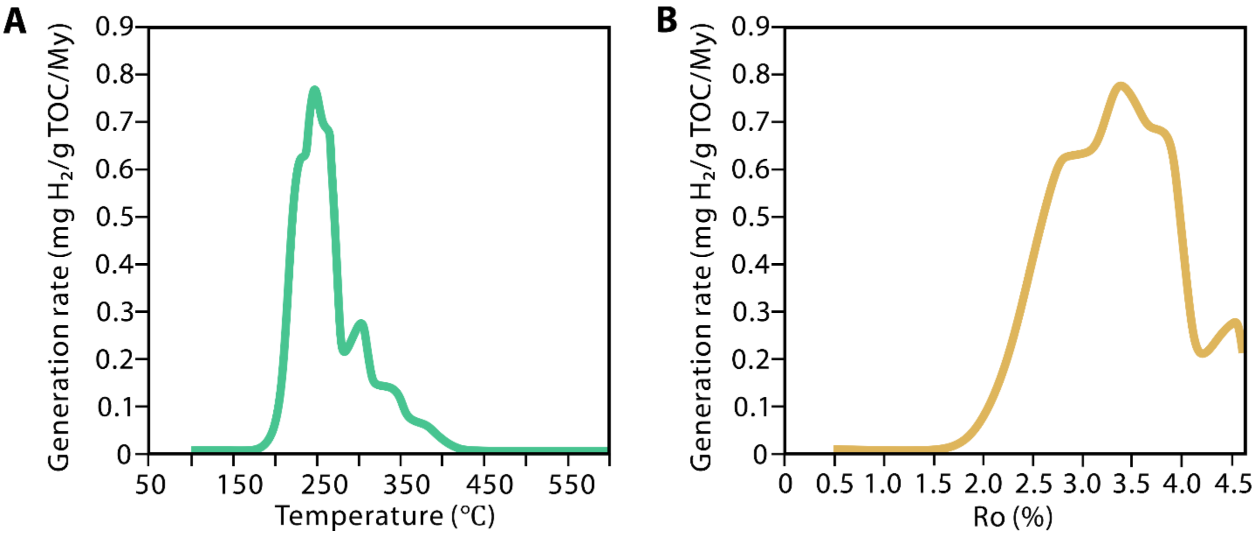

Fig. S8. Generation of hydrogen from sedimentary organic matter during thermal evolution through kinetic simulations, modified from (*55*). (A) The variation characteristics of natural hydrogen generation rate with increasing temperature. (B) The variation characteristics of natural hydrogen generation rate with increasing vitrinite reflectance (R_o).

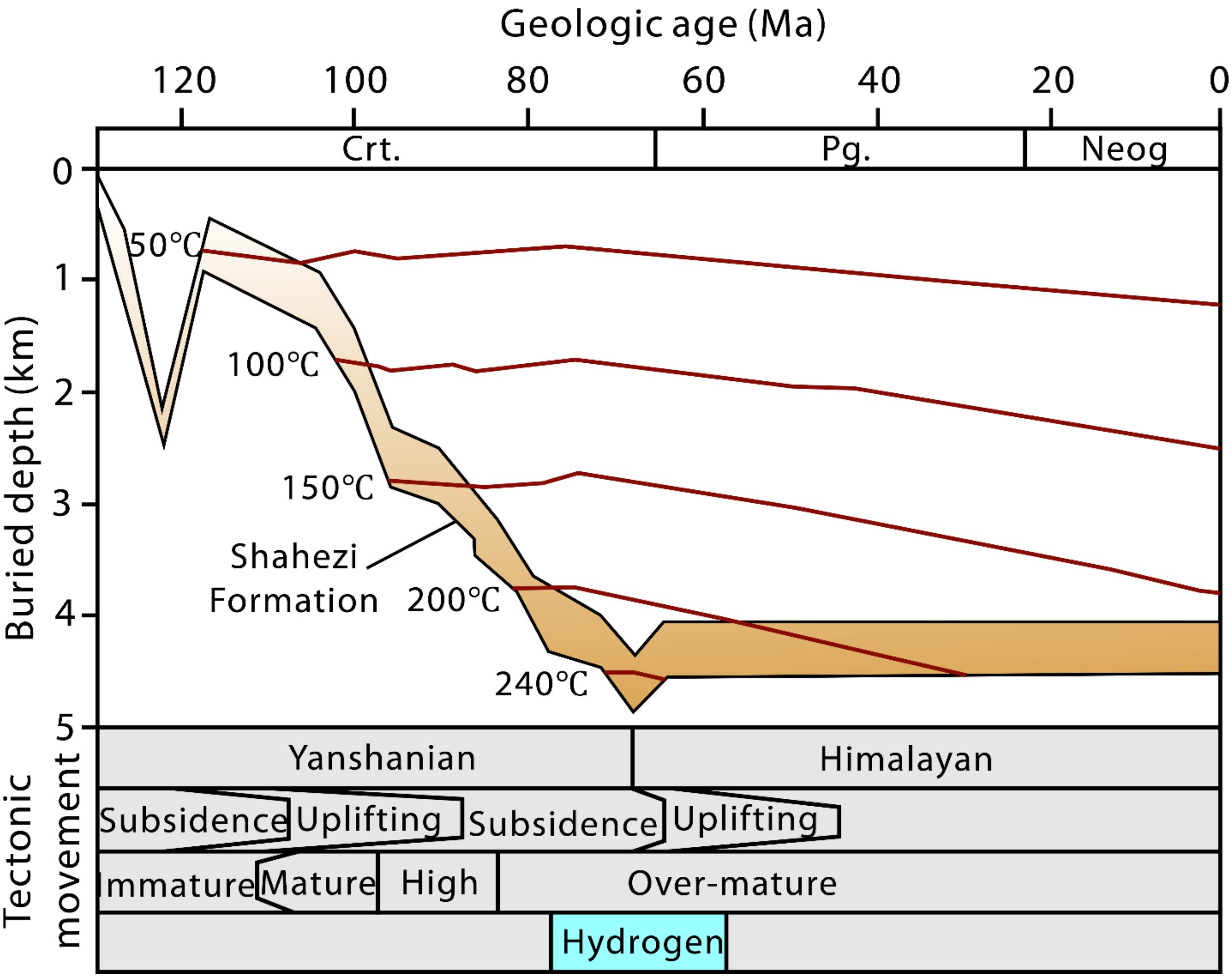

Fig. S9. Tectonic burial history of the Shahezi Formation shale suggests that it may have generated natural hydrogen during over-mature stage, modified from (*101*).

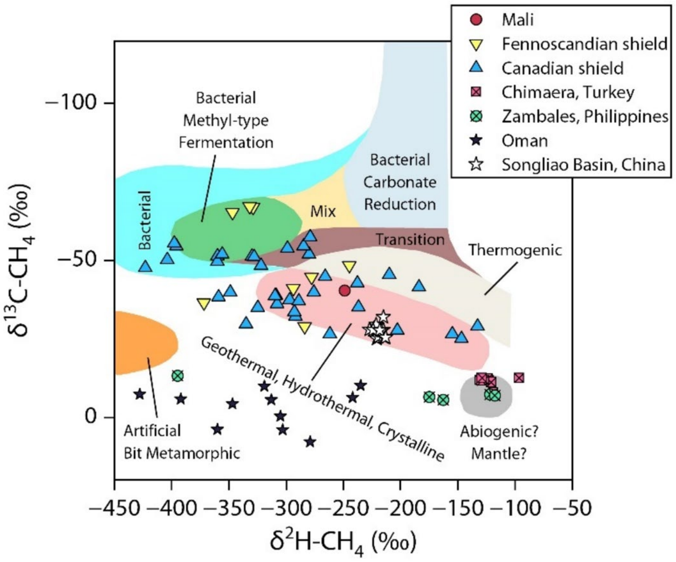

Fig. S10. Origin of natural gas indicated by carbon and hydrogen isotopes of CH_4 component, modified from (*102*). CH_4 in Oman (*10*), Zambales (*10, 92*), and Chimaera (*6, 103*) is typically of abiotic origin. CH_4 in the Songliao Basin, Mali (*17*), and shields (*104, 105*) is of mixed origin.

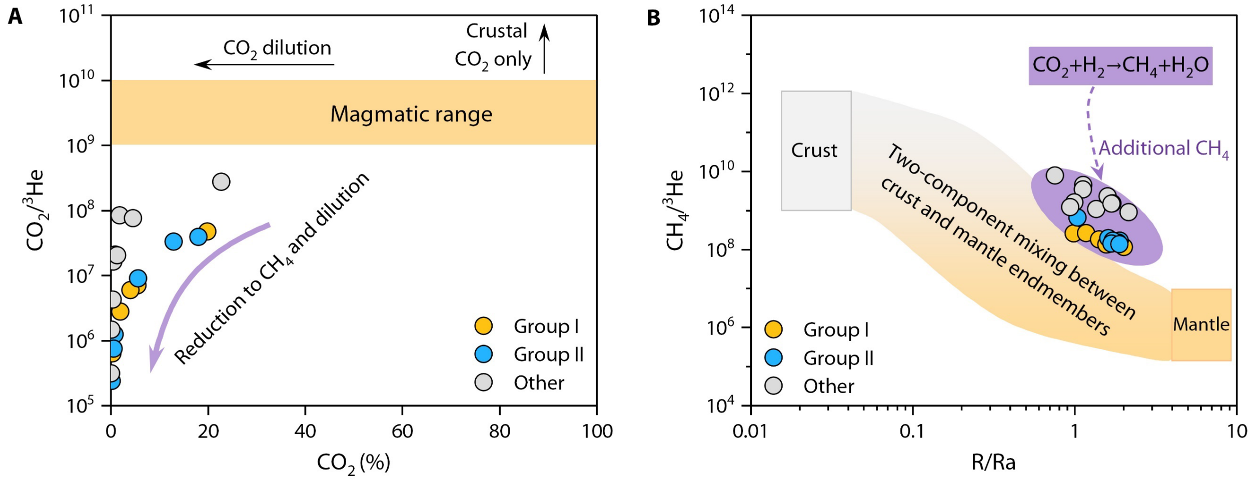

Fig. S11. Plot of CO_2 contents versus $CO_2/^3He$ values (A) and $CH_4/^3He$ values (B) with respect to mixing lines between crustal and mantle end-members, modified from (*64, 106*). The yellow-shaded region in (A) is the magmatic range as defined by (*107*).

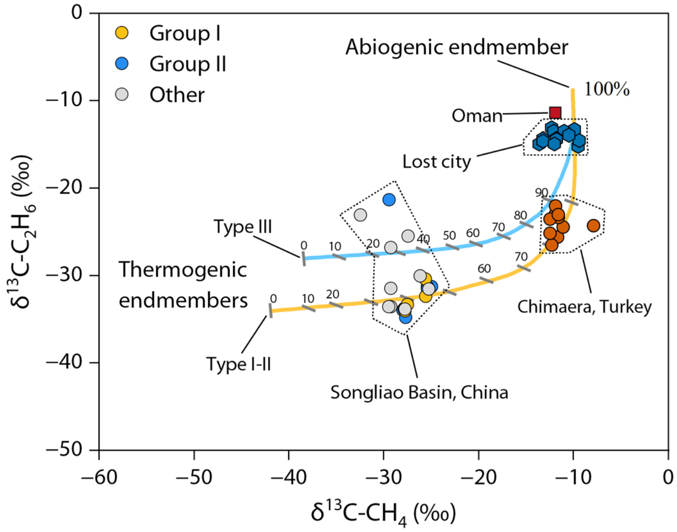

Fig. S12. The $\delta^{13}C_1$ vs. $\delta^{13}C_2$ plot with mixing lines between two thermogenic endmembers and the abiotic endmember, modified from (*6*).

Table S1. Chemical and isotopic composition of natural gas from theYingcheng Formation, Songliao Basin, China. The results are reported in δ notations relative to Vienna Pee Dee Belemnite (VPDB) for carbon and Vienna Standard Mean Ocean Water (VSMOW) for hydrogen, respective analytical uncertainties being of ±0.3‰ and ±3‰.

Well	Depth (m)	Gas composition (%)							$\delta^{13}C$ (‰, VPDB)				$\delta^{2}H$ (‰, VSMOW)				R/Ra
		He	H_2	N_2	CO_2	CH_4	C_2H_6	C_3H_8	CH_4	C_2H_6	C_3H_8	CO_2	H_2	CH_4	C_2H_6	C_3H_8	
DS3	3306	0.0099	1.26	1.18	0.0018	96.46	2.06	0.21	-29.4	-21.3	-23.8	/	-556	-222	-229	/	1.04
ZS16-1	3646	0.021	0.15	1.95	0.70	94.23	2.15	0.47	-25.0	-31.3	-32.8	-11.6	-522	-221	-209	/	1.89
ZS16-P2	3910	0.027	0.25	1.67	5.43	89.74	1.69	0.24	-25.6	-30.4	-30.8	-7.3	-636	-220	-192	/	2.01
XS8-305	3774	0.017	0.28	8.61	12.87	75.41	2.14	0.76	-28.0	-33.9	-33.8	-6.8	-573	-227	-220	-182	1.61
ZS19-P1S	4041	0.027	0.0025	1.36	22.68	74.61	1.16	0.074	-26.2	-30.0	/	-5.8	/	/	/	/	2.15
XS1-304	3557	0.013	0.029	1.19	1.74	93.56	2.37	0.41	-29.3	-33.5	-34.7	-3.9	/	/	/	/	1.12
XS14	3798	0.026	0.23	1.36	0.23	94.17	3.01	0.58	-25.6	-32.4	-33.2	/	-608	-215	-196	-163	0.98
XS1-203	3539	0.018	0.005	1.26	0.85	94.64	2.44	0.44	-29.5	-33.5	-34.7	-6.0	/	/	/	/	1.60
W24-13	1942	0.045	0	3.50	0.027	94.26	1.73	0.24	-29.3	-26.8	-24.9	/	/	/	/	/	1.35
DS17	2978	0.011	0.0021	0.53	0.21	92.25	5.04	0.77	-32.5	-23.1	-20.8	/	/	/	/	/	0.75
SSCP102	3449	0.021	0.15	2.16	0.015	91.59	4.61	1.06	/	-24.3	-27.5	/	-621	-215	-188	-178	1.17
XS6	3566	0.017	0.002	1.34	0.44	95.04	2.47	0.39	-29.3	-31.5	-32.3	-11.8	/	/	/	/	1.12
ZS16-P3	3970	0.024	0.73	1.55	0.15	94.99	2.24	0.53	-25.4	-31.2	-32.7	/	-578	-214	-178	-162	1.85
XS8-GP2	4228	0.019	0.63	1.60	17.98	77.70	1.95	0.45	-27.9	-33.8	-33.4	-7.4	-557	-215	-180	-176	1.71
SS1	2474	0.041	0.062	3.13	0.085	93.92	2.25	0.33	-27.5	-25.4	-24.0	/	/	/	/	/	0.99
XS8-GP1	4177	0.021	5.00	1.49	19.83	75.96	1.96	0.48	-27.8	-33.8	-34.1	-8.5	-698	-224	-225	-162	1.41
XS903	3817	0.026	0.16	2.15	5.58	88.63	2.03	0.32	-27.7	-34.8	-33.7	-6.5	-557	-218	-189	/	1.68
ZS16	3419	0.025	0.042	1.42	1.23	92.64	2.14	0.49	-25.3	-31.5	-33.2	-8.7	/	/	/	/	1.70
SS103H	3847	0.060	0.072	2.34	0.34	97.09	0.084	0.026	-32.3	/	/	/	/	/	/	/	0.94
XS3-P2	4630	0.030	1.20	2.26	1.89	92.96	2.17	0.35	-27.5	-33.3	-32.5	-12.5	-665	-217	-204	/	1.60
XS901	3759	0.030	1.61	2.51	3.96	90.69	2.15	0.40	-27.8	-34.0	-33.2	-9.0	-650	-218	-163	/	1.56
XS902	3774	0.025	0.026	2.36	4.50	88.99	2.14	0.42	-27.8	-33.8	-34.3	-6.3	/	/	/	/	1.68
ZS16-P1	4041	0.026	0.33	1.57	0.52	95.25	2.00	0.34	-25.4	-31.5	-32.7	-13.2	-567	-213	-191	/	1.88

Table S2. Natural hydrogen production from different origins, hydrogen reserves, and hydrogen store rate in the Qingshen gas deposit of the Songliao Basin.

Natural hydrogen production from different origins (m^3)			Hydrogen reserves (m^3)		Hydrogen store rate in natural gas systems	
Radiolytic hydrogen	WRI hydrogen	Mantle-derived hydrogen	Original	Current	Original	Current
572×10^9	248×10^9	127×10^9	62.4×10^9	13.4×10^9	6.6%	1.4%

REFERENCES AND NOTES

1. IEA, “The Future of Hydrogen” (2019); https://iea.org/reports/the-future-of-hydrogen.

2. M. Liebreich, “The Clean Hydrogen Ladder [Now updated to V4.1]” (Liebreich Associates, 2021); https://liebreich.com/the-clean-hydrogen-ladder-now-updated-to-v4-1/.

3. E. Hand, Hidden hydrogen: Earth may hold vast stores of a renewable, carbon-free fuel? *Science* **379**, 630–636 (2023).

4. F. Osselin, C. Soulaine, C. Fauguerolles, E. C. Gaucher, B. Scaillet, M. Pichavant, Orange hydrogen is the new green. *Nat. Geosci.* **15**, 765–769 (2022).

5. B. Sherwood Lollar, T. C. Onstott, G. Lacrampe-Couloume, C. J. Ballentine, The contribution of the Precambrian continental lithosphere to global H_2 production. *Nature* **516**, 379–382 (2014).

6. G. Etiope, M. Schoell, H. Hosgörmez, Abiotic methane flux from the Chimaera seep and Tekirova ophiolites (Turkey): Understanding gas exhalation from low temperature serpentinization and implications for Mars. *Earth Planet. Sci. Lett.* **310**, 96–104 (2011).

7. J. Guélard, V. Beaumont, V. Rouchon, F. Guyot, D. Pillot, D. Jézéquel, M. Ader, K. D. Newell, E. Deville, Natural H_2 in Kansas: Deep or shallow origin? *Geochem. Geophys. Geosyst.* **18**, 1841–1865 (2017).

8. C. Neal, G. Stanger, Hydrogen generation from mantle source rocks in Oman. *Earth Planet. Sci. Lett.* **66**, 315–320 (1983).

9. L. Truche, F.-V. Donzé, E. Goskolli, B. Muceku, C. Loisy, C. Monnin, H. Dutoit, A. Cerepi, A deep reservoir for hydrogen drives intense degassing in the Bulqizë ophiolite. *Science* **383**, 618–621 (2024).

10. C. Vacquand, E. Deville, V. Beaumont, F. Guyot, O. Sissmann, D. Pillot, C. Arcilla, A. Prinzhofer, Reduced gas seepages in ophiolitic complexes: Evidences for multiple origins of the H_2-CH_4-N_2 gas mixtures. *Geochim. Cosmochim. Acta* **223**, 437–461 (2018).

11. V. Zgonnik, The occurrence and geoscience of natural hydrogen: A comprehensive review. *Earth Sci. Rev.* **203**, 103140 (2020).

12. Q. Liu, X. Wu, Q. Meng, D. Zhu, X. Huang, D. Zhu, P. Li, Z. Jin, Natural hydrogen: A potential carbon-free energy source. *Chin. Sci. Bull.* **69**, 2344–2350 (2024).

13. A. V. Milkov, Molecular hydrogen in surface and subsurface natural gases: Abundance, origins and ideas for deliberate exploration. *Earth Sci. Rev.* **230**, 104063 (2022).

14. C. J. Boreham, D. S. Edwards, K. Czado, N. Rollet, L. Wang, S. van der Wielen, D. Champion, R. Blewett, A. Feitz, P. A. Henson, Hydrogen in Australian natural gas: Occurrences, sources and resources. *APPEA J.* **61**, 163–191 (2021).

15. O. Jackson, S. R. Lawrence, I. P. Hutchinson, A. E. Stocks, A. C. Barnicoat, M. Powney, Natural hydrogen: Sources, systems and exploration plays. *Geoenergy* **2**, geoenergy2024–2002 (2024).

16. O. Maiga, E. Deville, J. Laval, A. Prinzhofer, A. B. Diallo, Trapping processes of large volumes of natural hydrogen in the subsurface: The emblematic case of the Bourakebougou H_2 field in Mali. *Int. J. Hydrogen Energy* **50**, 640–647 (2024).

17. A. Prinzhofer, C. S. Tahara Cissé, A. B. Diallo, Discovery of a large accumulation of natural hydrogen in Bourakebougou (Mali). *Int. J. Hydrogen Energy* **43**, 19315–19326 (2018).

18. G. Etiope, M. J. Whiticar, Abiotic methane in continental ultramafic rock systems: Towards a genetic model. *Appl. Geochem.* **102**, 139–152 (2019).

19. G. Proskurowski, M. D. Lilley, J. S. Seewald, G. L. Früh-Green, E. J. Olson, J. E. Lupton, S. P. Sylva, D. S. Kelley, Abiogenic hydrocarbon production at lost city hydrothermal field. *Science* **319**, 604–607 (2008).

20. Q. Williams, R. J. Hemley, Hydrogen in the deep Earth. *Annu. Rev. Earth Planet. Sci.* **29**, 365–418 (2001).

21. R. Karolytė, O. Warr, E. van Heerden, S. Flude, F. de Lange, S. Webb, C. J. Ballentine, B. Sherwood Lollar, The role of porosity in H_2/He production ratios in fracture fluids from the Witwatersrand Basin, South Africa. *Chem. Geol.* **595**, 120788 (2022).

22. O. Warr, T. Giunta, C. J. Ballentine, B. Sherwood Lollar, Mechanisms and rates of ^{4}He, ^{40}Ar, and H_2 production and accumulation in fracture fluids in Precambrian Shield environments. *Chem. Geol.* **530**, 119322 (2019).

23. M. Feng, M. J. An, H. S. Hou, T. Y. Fan, H. L. Zang, Channelised magma ascent and lithospheric zonation beneath the Songliao Basin, Northeast China, based on surface-wave tomography. *Tectonophysics* **862**, 229969 (2023).

24. Z. Li, J. Chen, H. Zou, C. Wang, Q. Meng, H. Liu, S. Wang, Mesozoic–Cenozoic tectonic evolution and dynamics of the Songliao Basin, NE Asia: Implications for the closure of the Paleo-Asian Ocean and Mongol-Okhotsk Ocean and subduction of the Paleo-Pacific Ocean. *Earth Sci. Rev.* **218**, 103471 (2021).

25. S. Han, Z. Tang, C. Wang, B. Horsfield, T. Wang, N. Mahlstedt, Hydrogen-rich gas discovery in continental scientific drilling project of Songliao Basin, Northeast China: New insights into deep Earth exploration. *Sci. Bull.* **67**, 1003–1006 (2022).

26. M. Schoell, Genetic characterization of natural gases. *AAPG Bull.* **67**, 2225–2238 (1983).

27. Y. Zou, Y. Cai, C. Zhang, X. Zhang, P. Peng, Variations of natural gas carbon isotope-type curves and their interpretation – A case study. *Org. Geochem.* **38**, 1398–1415 (2007).

28. H. Wycherley, A. Fleet, H. Shaw, Some observations on the origins of large volumes of carbon dioxide accumulations in sedimentary basins. *Mar. Pet. Geol.* **16**, 489–494 (1999).

29. B. Sherwood Lollar, K. Voglesonger, L. H. Lin, G. Lacrampe-Couloume, J. Telling, T. A. Abrajano, T. C. Onstott, L. M. Pratt, Hydrogeologic controls on episodic H_2 release from precambrian fractured rocks—Energy for deep subsurface life on Earth and Mars. *Astrobiology* **7**, 971–986 (2007).

30. E. R. Oxburgh, R. K. O'Nions, R. I. Hill, Helium isotopes in sedimentary basins. *Nature* **324**, 632–635 (1986).

31. D. W. Graham, Noble gas isotope geochemistry of mid-ocean ridge and ocean island basalts: Characterization of mantle source reservoirs. *Rev. Mineral. Geochem.* **47**, 247–317 (2002).

32. C. J. Ballentine, R. K. O'Nions, The nature of mantle neon contributions to Vienna Basin hydrocarbon reservoirs. *Earth Planet. Sci. Lett.* **113**, 553–567 (1992).

33. Q. Liu, X. Wu, X. Wang, Z. Jin, D. Zhu, Q. Meng, S. Huang, J. Liu, Q. Fu, Carbon and hydrogen isotopes of methane, ethane, and propane: A review of genetic identification of natural gas. *Earth Sci. Rev.* **190**, 247–272 (2019).

34. F. Robert, S. Derenne, G. Lombardi, K. Hassouni, A. Michau, P. Reinhardt, R. Duhamel, A. Gonzalez, K. Biron, Hydrogen isotope fractionation in methane plasma. *Proc. Natl. Acad. Sci. U.S.A.* **114**, 870–874 (2017).

35. Q. Liu, X. Wu, X. Huang, D. Zhu, Q. Meng, D. Zhu, H. Xu, J. Liu, P. Li, Z. Zhou, K. Zhang, Z. Jin, Integrated geochemical identification of natural hydrogen sources. *Sci. Bull.* **24**, S2095–9273 (2024).

36. F. Beulig, F. Schubert, R. R. Adhikari, C. Glombitza, V. B. Heuer, K. U. Hinrichs, K. L. Homola, F. Inagaki, B. B. Jørgensen, J. Kallmeyer, S. J. E. Krause, Y. Morono, J. Sauvage, A. J. Spivack, T. Treude, Rapid metabolism fosters microbial survival in the deep, hot subseafloor biosphere. *Nat. Commun.* **13**, 312 (2022).

37. I. M. Head, D. M. Jones, S. R. Larter, Biological activity in the deep subsurface and the origin of heavy oil. *Nature* **426**, 344–352 (2003).

38. N. J. Pester, M. E. Conrad, K. G. Knauss, D. J. DePaolo, Kinetics of D/H isotope fractionation between molecular hydrogen and water. *Geochim. Cosmochim. Acta* **242**, 191–212 (2018).

39. A. Ricci, B. I. Kleine, J. Fiebig, J. Gunnarsson-Robin, K. Mativo Kamunya, B. Mountain, A. Stefánsson, Equilibrium and kinetic controls on molecular hydrogen abundance and hydrogen isotope fractionation in hydrothermal fluids. *Earth Planet. Sci. Lett.* **579**, 117338 (2022).

40. Y. Horibe, H. Craig, DH fractionation in the system methane-hydrogen-water. *Geochim. Cosmochim. Acta* **59**, 5209–5217 (1995).

41. G. Proskurowski, M. D. Lilley, D. S. Kelley, E. J. Olson, Low temperature volatile production at the Lost City Hydrothermal Field, evidence from a hydrogen stable isotope geothermometer. *Chem. Geol.* **229**, 331–343 (2006).

42. C. Liu, E. Nicotra, X. Shan, J. Yi, G. Ventura, The Cretaceous volcanism of the Songliao Basin: Mantle sources, magma evolution processes and implications for the NE China geodynamics - A review. *Earth Sci. Rev.* **237**, 104294 (2023).

43. S. Han, C. Xiang, X. Du, L. Xie, J. Huang, C. Wang, Geochemistry and origins of hydrogen-containing natural gases in deep Songliao Basin, China: Insights from continental scientific drilling. *Pet. Sci.* **21**, 741–751 (2024).

44. G. Wang, W. Lin, F. Liu, H. Gan, S. Wang, G. Yue, X. Long, Y. Liu, Theory and survey practice of deep heat accumulation in geothermalsystem and exploration practice. *Acta Geol. Sin.* **97**, 639–660 (2023).

45. L. Truche, F. Bourdelle, S. Salvi, N. Lefeuvre, A. Zug, E. Lloret, Hydrogen generation during hydrothermal alteration of peralkaline granite. *Geochim. Cosmochim. Acta* **308**, 42–59 (2021).

46. J. R. Smyth, D. J. Frost, F. Nestola, C. M. Holl, G. Bromiley, Olivine hydration in the deep upper mantle: Effects of temperature and silica activity. *Geophys. Res. Lett.* **33**, L15301 (2006).

47. S. Demouchy, N. Bolfan-Casanova, Distribution and transport of hydrogen in the lithospheric mantle: A review. *Lithos* **240-243**, 402–425 (2016).

48. A. S. Merdith, I. Daniel, D. Sverjensky, M. Andreani, B. Mather, S. Williams, A. Vitale Brovarone, Global hydrogen production during high-pressure serpentinization of subducting slabs. *Geochem. Geophys. Geosyst.* **24**, e2023GC010947 (2023).

49. H. Liu, B. Zhang, H. Fei, L. Liu, A first-principles molecular dynamics study of molecular hydrogen diffusion in Fe-free olivine. *Geosci. Front.* **16**, 101926 (2025).

50. B. H. Lodhia, L. Peeters, E. Frery, A review of the migration of hydrogen from the planetary to basin scale. *J. Geophys. Res. Solid Earth* **129**, e2024JB028715 (2024).

51. T. P. Fischer, G. Chiodini, “Chapter 45 - Volcanic, magmatic and hydrothermal gases” in *The Encyclopedia of Volcanoes* (Second Edition), H. Sigurdsson, Ed. (Academic Press, 2015), pp. 779–797.

52. Z. Jin, W. Hu, L. Zhang, M. Tao, *Deep-derived Fluid and its Effect on Hydrocarbon Cccumulation* (Science Press, Beijing, 2007).

53. Q. Meng, Y. Sun, J. Tong, Q. Fu, J. Zhu, D. Zhu, Z. Jin, Distribution and geochemical characteristics of hydrogen in natural gas from the jiyang depression, Eastern China. *Acta Geol. Sin. (Engl. Ed.)* **89**, 1616–1624 (2015).

54. D. V. Bekaert, S. J. Turner, M. W. Broadley, J. D. Barnes, S. A. Halldórsson, J. Labidi, J. Wade, K. J. Walowski, P. H. Barry, Subduction-driven volatile recycling: A global mass balance. *Annu. Rev. Earth Planet. Sci.* **49**, 37–70 (2021).

55. B. Horsfield, N. Mahlstedt, P. Weniger, D. Misch, S. Vranjes-Wessely, S. Han, C. Wang, Molecular hydrogen from organic sources in the deep Songliao Basin, P.R. China. *Int. J. Hydrogen Energy* **47**, 16750–16774 (2022).

56. X. Li, J. Horita, Kinetic and equilibrium reactions on natural and laboratory generation of thermogenic gases from Type II marine shale. *Geochim. Cosmochim. Acta* **333**, 263–283 (2022).

57. A. Schimmelmann, M. D. Lewan, R. P. Wintsch, D/H isotope ratios of kerogen, bitumen, oil, and water in hydrous pyrolysis of source rocks containing kerogen types I, II, IIS, and III. *Geochim. Cosmochim. Acta* **63**, 3751–3766 (1999).

58. M. N. Abu Tahari, F. Salleh, T. S. Tengku Saharuddin, A. Samsuri, S. Samidin, M. A. Yarmo, Influence of hydrogen and carbon monoxide on reduction behavior of iron oxide at high temperature: Effect on reduction gas concentrations. *Int. J. Hydrogen Energy* **46**, 24791–24805 (2021).

59. S. Lu, J. Li, H. Xue, F. Chen, Q. Xu, M. Wang, W. Li, X. Pang, Pyrolytic gaseous hydrocarbon generation and the kinetics of carbon isotope fractionation in representative model compounds with different chemical structures. *Geochem. Geophys. Geosyst.* **20**, 1773–1793 (2019).

60. B. Sherwood Lollar, T. D. Westgate, J. A. Ward, G. F. Slater, G. Lacrampe-Couloume, Abiogenic formation of alkanes in the Earth's crust as a minor source for global hydrocarbon reservoirs. *Nature* **416**, 522–524 (2002).

61. H. P. Scott, R. J. Hemley, H.-k. Mao, D. R. Herschbach, L. E. Fried, W. M. Howard, S. Bastea, Generation of methane in the Earth's mantle: In situ high pressure–temperature measurements of carbonate reduction. *Proc. Natl. Acad. Sci. U.S.A.* **101**, 14023–14026 (2004).

62. A. Kolesnikov, V. G. Kutcherov, A. F. Goncharov, Methane-derived hydrocarbons produced under upper-mantle conditions. *Nat. Geosci.* **2**, 566–570 (2009).

63. G. Etiope, B. Sherwood Lollar, Abiotic methane on Earth. *Rev. Geophys.* **51**, 276–299 (2013).

64. Q. Liu, J. Dai, Z. Jin, J. Li, X. Wu, Q. Meng, C. Yang, Q. Zhou, Z. Feng, D. Zhu, Abnormal carbon and hydrogen isotopes of alkane gases from the Qingshen gas field, Songliao Basin, China, suggesting abiogenic alkanes? *J. Asian Earth Sci.* **115**, 285–297 (2016).

65. J. Horita, M. E. Berndt, Abiogenic methane formation and isotopic fractionation under hydrothermal conditions. *Science* **285**, 1055–1057 (1999).

66. A. Cheng, B. Sherwood Lollar, J. G. Gluyas, C. J. Ballentine, Primary N_2–He gas field formation in intracratonic sedimentary basins. *Nature* **615**, 94–99 (2023).

67. L.-H. Lin, J. Hall, J. Lippmann-Pipke, J. A. Ward, B. Sherwood Lollar, M. DeFlaun, R. Rothmel, D. Moser, T. M. Gihring, B. Mislowack, T. C. Onstott, Radiolytic H_2 in continental crust: Nuclear power for deep subsurface microbial communities. *Geochem. Geophys. Geosyst.* **6**, Q07003 (2005).

68. L.-H. Lin, G. F. Slater, B. Sherwood Lollar, G. Lacrampe-Couloume, T. C. Onstott, The yield and isotopic composition of radiolytic H_2, a potential energy source for the deep subsurface biosphere. *Geochim. Cosmochim. Acta* **69**, 893–903 (2005).

69. Z. Feng, C. Yin, J. Qi, J. Dong, Main factors controlling hydrocarbon accumulation in large volcanic gas fields: A case study of the Qingshen gas field in the Songliao basin. *Acta Petrol. Sin.* **26**, 21–32 (2010).

70. N. H. Sleep, D. K. Bird, Niches of the pre-photosynthetic biosphere and geologic preservation of Earth's earliest ecology. *Geobiology* **5**, 101–117 (2007).

71. T. Wang, C. Wang, J. Ramezani, X. Wan, Z. Yu, Y. Gao, H. He, H. Wu, High-precision geochronology of the Early Cretaceous Yingcheng Formation and its stratigraphic implications for Songliao Basin, China. *Geosci. Front.* **13**, 101386 (2022).

72. L. Zhang, Z. Wang, B. Xu, H. Zou, P. Zhao, H. Zhang, Neoproterozoic–Early Cambrian igneous and sedimentary sequences in the Songliao Block, NE China: Records of Rodinia supercontinent evolution in eastern Central Asian orogenic Belt. *Precambrian Res.* **381**, 106865 (2022).

73. F. Gaillard, M. A. Bouhifd, E. Füri, V. Malavergne, Y. Marrocchi, L. Noack, G. Ortenzi, M. Roskosz, S. Vulpius, The diverse planetary ingassing/outgassing paths produced over billions of years of magmatic activity. *Space Sci. Rev.* **217**, 22 (2021).

74. B. Marty, G. Avice, Y. Sano, K. Altwegg, H. Balsiger, M. Hässig, A. Morbidelli, O. Mousis, M. Rubin, Origins of volatile elements (H, C, N, noble gases) on Earth and Mars in light of recent results from the ROSETTA cometary mission. *Earth Planet. Sci. Lett.* **441**, 91–102 (2016).

75. Y. Shuai, G. Etiope, S. C. Zhang, P. M. J. Douglas, L. Huang, J. M. Eiler, Methane clumped isotopes in the Songliao Basin (China): New insights into abiotic vs. biotic hydrocarbon formation. *Earth Planet. Sci. Lett.* **482**, 213–221 (2018).

76. F. Zhang, H. Chen, X. Yu, C. Dong, S. Yang, Y. Pang, G. E. Batt, Early Cretaceous volcanism in the northern Songliao Basin, NE China, and its geodynamic implication. *Gondw. Res.* **19**, 163–176 (2011).

77. Y. Hao, Z. Pang, J. Tian, Y. Wang, Z. Li, L. Li, L. Xing, Origin and evolution of hydrogen-rich gas discharges from a hot spring in the eastern coastal area of China. *Chem. Geol.* **538**, 119477 (2020).

78. K. Suda, Y. Ueno, M. Yoshizaki, H. Nakamura, K. Kurokawa, E. Nishiyama, K. Yoshino, Y. Hongoh, K. Kawachi, S. Omori, K. Yamada, N. Yoshida, S. Maruyama, Origin of methane in serpentinite-hosted hydrothermal systems: The CH_4–H_2–H_2O hydrogen isotope systematics of the Hakuba Happo hot spring. *Earth Planet. Sci. Lett.* **386**, 112–125 (2014).

79. W. Zhao, Z. Guo, M. Lei, M. Zhang, L. Ma, D. Fortin, G. Zheng, Volcanogenic CO_2 degassing in the songliao continental rift system, NE China. *Geofluids* **2019**, 1–14 (2019).

80. X. Liu, X. Fu, D. Liu, W. Wei, X. Lu, C. Liu, W. Wang, H. Gao, Distribution of mantle-derived CO_2 gas reservoir and its relationship with basement faults in Songliao Basin, China. *J. Nat. Gas Sci. Eng.* **56**, 593–607 (2018).

81. Y. Liu, S. Xie, G. Feng, C. Su, Q. Xu, T. Gao, Evolution of kerogen structure during the carbonization stage. *Org. Geochem.* **190**, 104743 (2024).

82. J. Dai, J. Li, X. Luo, W. Zhang, G. Hu, C. Ma, J. Guo, S. Ge, Stable carbon isotope compositions and source rock geochemistry of the giant gas accumulations in the Ordos Basin, China. *Org. Geochem.* **36**, 1617–1635 (2005).

83. J. A. Welhan, H. Craig, Methane and hydrogen in East Pacific Rise hydrothermal fluids. *Geophys. Res. Lett.* **6**, 829–831 (1979).

84. J. Dai, C. Zou, S. Zhang, J. Li, Y. Ni, G. Hu, X. Luo, S. Tao, G. Zhu, J. Mi, Z. Li, A. Hu, C. Yang, Q. Zhou, Y. Shuai, Y. Zhang, C. Ma, Discrimination of abiogenic and biogenic alkane gases. *Sci. China, Ser. D: Earth Sci.* **51**, 1737–1749 (2008).

85. Y. Li, R. Gao, J. Yao, S. Mi, W. Li, X. Xiong, J. Gao, The crust velocity structure of Da Hinggan Ling orgenic belt and the basins on both sides. *Prog. Geophys.* **29**, 73–83 (2014).

86. Z. Li, Y. Yan, Characteristics of radiogenic heat of the Jurassic in Songliao Basin and its significance. *Geotecton. Metallog.* **26**, 297–299 (2002).

87. S. Zhang, C. Zou, C. Peng, J. Zhao, N. Li, X. Zhang, H. Ma, Y. Niu, Abnormally high natural radioactivity zones in the main borehole of the Continental Scientific Drilling Project of Cretaceous Songliao Basin: Geophysical log responses and genesis analysis. *Chin. J. Geophys.* **61**, 4712–4728 (2018).

88. S. Dai, L. Wang, Z. Xin, Testing and characteristic analysis of elastic parameters of basement rock in the paleo-central uplift belt in the northern Songliao Basin. *Oil Geophys. Prospect.* **58**, 443–453 (2023).

89. C. M. Bethke, A numerical model of compaction-driven groundwater flow and heat transfer and its application to the paleohydrology of intracratonic sedimentary basins. *J. Geophys. Res. Solid Earth* **90**, 6817–6828 (1985).

90. S. Gao, T. Luo, B. Zhang, H. Zhang, Y. Han, Z. Zhao, H. Kern, Structure and composition of the continental crust in East China. *Sci. China, Ser. D: Earth Sci.* **42**, 129–140 (1999).

91. R. M. Coveney, E. D. Goebel, E. J. Zeller, G. A. M. Dreschhoff, E. E. Angino, Serpentinization and the origin of hydrogen gas in Kansas. *AAPG Bull.* **71**, 39–48 (1987).

92. T. A. Abrajano, N. C. Sturchio, J. K. Bohlke, G. L. Lyon, R. J. Poreda, C. M. Stevens, Methane-hydrogen gas seeps, Zambales Ophiolite, Philippines: Deep or shallow origin? *Chem. Geol.* **71**, 211–222 (1988).

93. Z. Shangguan, W. Huo, δD values of escaped H_2 from hot springs at the Tengchong Rehai geothermal area and its origin. *Chin. Sci. Bull.* **47**, 148–150 (2002).

94. Y. Shuai, S. Zhang, A. Su, H. Wang, B. Cai, H. Wang, Geochemical evidence for strong ongoing methanogenesis in Sanhu region of Qaidam Basin. *Sci. China Ser. D: Earth Sci.* **53**, 84–90 (2009).

95. G. Etiope, N. Samardžić, F. Grassa, H. Hrvatović, N. Miošić, F. Skopljak, Methane and hydrogen in hyperalkaline groundwaters of the serpentinized Dinaride ophiolite belt, Bosnia and Herzegovina. *Appl. Geochem.* **84**, 286–296 (2017).

96. N. Suzuki, H. Saito, T. Hoshino, Hydrogen gas of organic origin in shales and metapelites. *Int. J. Coal Geol.* **173**, 227–236 (2017).

97. X. Zhai, L. Yang, X. Xue, Y. Gao, P. Wang, Prediction of the bottom hole geotemperature, formation pressure and formation fracture pressure of the Continental Scientific Drilling of Cretaceous Songliao Basin (SK2). *Earth Sci. Front.* **24**, 257–264 (2017).

98. J. Han, Z. Guo, W. Liu, H. Hou, G. Liu, S. Han, L. Liu, T. Wang, Deep dynamic process of lithosphere thinning in Songliao basin. *Chin. J. Geophys.* **61**, 2265–2279 (2018).

99. Y. Hao, X. Kuang, Y. Feng, Y. Wang, H. Zhou, C. Zheng, Discovery and genesis of helium-rich geothermal fluids along the India–Asia continental convergent margin. *Geochim. Cosmochim. Acta* **360**, 175–191 (2023).

100. J. Dai, G. Hu, Y. Ni, J. Li, X. Luo, C. Yang, A. Hu, Q. Zhou, Natural gas accumulation in Eastern China. *Energy Explor. Exploit.* **27**, 225–259 (2009).

101. D. Xiao, S. Lu, M. Shao, N. Zhou, R. Zhao, Y. Peng, Comparison of marine and continental shale gas reservoirs and their gas-bearing properties in China: The examples of the longmaxi and shahezi shales. *Energy Fuel* **35**, 4029–4043 (2021).

102. M. J. Whiticar, Carbon and hydrogen isotope systematics of bacterial formation and oxidation of methane. *Chem. Geol.* **161**, 291–314 (1999).

103. H. Hosgormez, G. Etiope, M. N. YalÇIn, New evidence for a mixed inorganic and organic origin of the Olympic Chimaera fire (Turkey): A large onshore seepage of abiogenic gas. *Geofluids* **8**, 263–273 (2008).

104. B. Sherwood Lollar, S. K. Frape, P. Fritz, S. A. Macko, J. A. Welhan, R. Blomqvist, P. W. Lahermo, Evidence for bacterially generated hydrocarbon gas in Canadian shield and fennoscandian shield rocks. *Geochim. Cosmochim. Acta* **57**, 5073–5085 (1993).

105. B. Sherwood Lollar, S. K. Frape, S. M. Weise, P. Fritz, S. A. Macko, J. A. Welhan, Abiogenic methanogenesis in crystalline rocks. *Geochim. Cosmochim. Acta* **57**, 5087–5097 (1993).

106. Y. Sano, B. Marty, Origin of carbon in fumarolic gas from island arcs. *Chem. Geol.* **119**, 265–274 (1995).

107. B. Marty, A. Jambon, C^3He in volatile fluxes from the solid Earth: implications for carbon geodynamics. *Earth Planet. Sci. Lett.* **83**, 16–26 (1987).
